# Supplementary material for: Patterns of Positive Selection in Seven Ant Genomes
Source: Mol Biol Evol. 2014 Apr 29;31(7):1661–85. doi: 10.1093/molbev/msu141 (PMC4069625; doi:10.1093/molbev/msu141)
Supplement: Supplementary Data [file supp_31_7_1661__index.html]

Patterns of positive selection in seven ant genomes — Patterns of Positive Selection in Seven Ant Genomes — Patterns of Positive Selection in Seven Ant Genomes — Supplementary Data 

# Patterns of Positive Selection in Seven Ant Genomes

## Supplementary Data

files

**Files in this Data Supplement:**

- Supplementary Data - pdf file
- Supplementary Data - pdf file
- Supplementary Data - xls file
