# Supplementary material for: Patterns of Positive Selection in Seven Ant Genomes
Source: Mol Biol Evol. 2014 Apr 29;31(7):1661–85. doi: 10.1093/molbev/msu141 (PMC4069625; doi:10.1093/molbev/msu141)

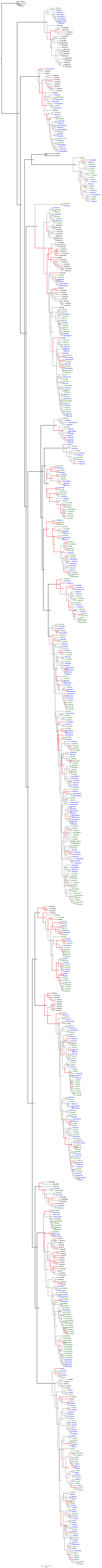

Supplement: Supplementary Data [file supp_msu141_Figure_S1.pdf]
