# Supplementary material for: Patterns of Positive Selection in Seven Ant Genomes
Source: Mol Biol Evol. 2014 Apr 29;31(7):1661–85. doi: 10.1093/molbev/msu141 (PMC4069625; doi:10.1093/molbev/msu141)
Supplement: Supplementary Data [file supp_msu141_Roux_et_al_sup_information.pdf]

Supplementary information for:

Patterns of positive selection in seven ant genomes

Julien Roux[1,2,5,7], Eyal Privman[1,2,6], Sébastien Moretti[1,2,3], Josephine T. Daub[1,2,4], Marc Robinson-Rechavi[1,2], Laurent Keller[1]

[1] Department of Ecology and Evolution, University of Lausanne, 1015 Lausanne, Switzerland

[2] SIB Swiss Institute of Bioinformatics, 1015 Lausanne, Switzerland

[3] Vital-IT group, SIB Swiss Institute of Bioinformatics, 1015 Lausanne, Switzerland

[4] CMPG, Institute of Ecology and Evolution, University of Bern, Baltzerstrasse 6, 3012 Bern, Switzerland

[5] Present address: Department of Human Genetics, University of Chicago, Chicago, IL 60637, USA

[6] Present address: Department of Evolutionary and Environmental Biology, University of Haifa, Israel

[7] Corresponding author: Julien.Roux@unil.ch

Table of Content

Supplementary text 3

Potential sources of false positives in the scan for positive selection 3

Validation with the site test of Codeml 5

Influence of false positives on the gene set enrichment approach 6

Gene set enrichment test with KEGG pathways 7

Characterization of functional biases in the datasets 7

Comparison with other lineages 9

Examples of Codeml control files for the branch-site test 11

Alternative hypothesis 11

Null model 11

Examples of Codeml control files for the site test 12

Alternative hypothesis (M8) 12

Null model (M8a) 12

Newick files for 16 olfactory receptors subtrees 13

Figure S1 22

Figure S2 22

Figure S3 23

Figure S4 24

Figure S5 25

Figure S6 26

Figure S7 26

References 28

Supplementary text

Potential sources of false positives in the scan for positive selection

Phylogenetic tests of positive selection in protein-coding genes usually contrast substitution rates at non-synonymous sites to substitution rates at synonymous sites, taken as a proxy to neutral rates of evolution. However a number of species are known to experience selective constraints at synonymous sites, potentially leading to false positives (Yang, dos Reis 2011). For example in most species of the *Drosophila* phylogeny, synonymous codons are optimized for better protein translation (Drosophila 12 Genomes Consortium 2007). In our dataset, nine out of the twelve species studied (the seven ants, *Tribolium castaneum* and *Nasonia vitripennis*) display low codon usage bias with synonymous sites likely to behave as neutral sites (Simola et al. 2013). For all branches tested deeper in the phylogenetic tree (in red in Figure 1), the most parsimonious scenario is that they did not experience constraints at synonymous sites either.

Schneider *et al.* postulated that higher rate of detection on long branches of phylogenetic trees (Table 2) might be due to saturation of d_S, leading to overestimation of d_N/d_S (Schneider et al. 2009). Previous work has shown that the branch-site test should not suffer seriously from this problem if the number of species used is large enough and the phylogenetic depth reasonable (Studer, Robinson-Rechavi 2009; Fletcher, Yang 2010; Yang, dos Reis 2011). These conditions are met in our dataset, so d_S saturation should not be an important concern in our study. Simulations also showed that increasing branch lengths indeed does lead to saturation of d_S but also decreases the power of the test, making it unlikely to result in false positives (Gharib, Robinson-Rechavi 2013). When we applied a stringent filtering on our results, and did not consider all tests where d_S on the foreground branch was higher than 1, the percentage of families with significant signal for an episode of positive selection dropped to 24% (1,034 families), indicating that the majority of the positives cannot be caused by saturation of d_S. The drop was less severe when only ant branches were considered (from 20% to

15%; 620 families), indicating that d_S saturation is more likely to affect the longest branches of our phylogeny (branch #8 leading to hymenoptera and branch #7 leading to ants and bees) than the branches of the ant lineage.

Another source of false positives could be sequencing and assembly errors (Mallick et al. 2009; Schneider et al. 2009). These are particularly critical with second generation sequencing technologies used for most genomes of our dataset. However, these errors should not be shared between multiple species, so they are only expected to affect the results of tests on terminal branches of the tree. Among the two technologies used to sequence the 7 ant genomes, Roche 454 was shown to yield a higher rate of sequencing errors than Illumina (Luo et al. 2012), but we did not observe any obvious relation between the proportion of significant genes in terminal ant branches and the technology used to sequence the genomes (Roche 454, Illumina or a mix of both technologies; Kruskal-Wallis test, p = 0.32). When only internal branches of the phylogeny were considered, 1,481 families (35%) still displayed a significant signal for an episode of positive selection in any of the tested branches, and 267 families (6.3%) were significant on at least one the 6 internal branches of the ant lineage (197 of them – 74% – exclusively on these branches).

Finally, some studies have warned about the possibility that GC-biased gene conversion, a neutral process affecting genomes in very similar ways to positive selection, could lead to erroneous significance of the positive selection tests (Duret, Galtier 2009; Galtier et al. 2009; Ratnakumar et al. 2010). We have analyzed the G+C content at third codon positions (GC_3, more likely than first and second positions to reflect the action of neutral processes) of ant genes in families that had at least one branch significant in the ant lineage (FDR <= 0.1). We compared it to the GC_3 of genes in families which had a FDR of 1 in all ant branches, and are thus not likely to include false negatives. The distribution of GC_3 was not statistically different between the two groups (Kolmogorov-Smirnov test, p=0.27). We then calculated the variation in G+C content at third codon positions (ΔGC_3) along each branch of the families analyzed (see Materials and Methods). If GC-biased gene conversion was affecting the results of the branch-site test, we would expect a significant positive association between the score of the branch-site test and sudden shifts in G+C

content at third codon positions. But the correlation was very small and negative, between ΔGC_3 and the score of the branch-site test (ΔlnL) across all branches of the families tested (Spearman correlation $r=-0.045$, $p<10^{-15}$). If only branches which were significant at FDR=20% were considered, the correlation was much weaker ($r=-0.0011$, $p=0.95$). These result are consistent with a previous observation that ΔGC_3 explained a negligible amount of variation in the results of the branch-site test in vertebrate gene families (Studer et al. 2008). Thus, we are confident that the results from the branch-site test in ants are not strongly affected by potential false positives due to GC-biased gene conversion.

Validation with the site test of Codeml

To investigate how positive selection contributed to the evolution of large gene families, we applied the site test of Codeml on a more extensive dataset (not limited to single-copy orthologs, see Materials and Methods)(Yang et al. 2000). Not surprisingly, this yielded a much lower proportion of positives (11 families at 10% FDR; Table 1 and S19), because the site test detects families that experienced recurrent positive selection on the same sites on many branches of a phylogeny. This compares with only 22 single-ortholog gene families with significant signal for positive selection with the branch-site test in at least 4 different branches of their phylogenetic tree. Across gene families, there was a small but highly significant correlation between the site test scores (log-likelihood ratio) and the branch-site test scores (mean of transformed log-likelihood ratio, see Materials and Methods; Spearman correlation $\rho=0.13$, $p<10^{-15}$).

Finally, the gene set enrichment test applied on results of the site test confirmed the patterns observed with the branch-site test results, notably an enrichment of positive selection on mitochondrial functions (Table S13). Interestingly there was also a significant enrichment for functions related to amino acid transport, heme binding and detection of light stimulus involved in visual perception.

Influence of false positives on the gene set enrichment approach

The gene set enrichment test based on results of the positive selection test was designed to attenuate the influence of potential false positives (see Materials and Methods). We considered for each family a score reflecting how consistently positive selection was experienced on the different branches of the ant phylogeny (Table 3). The influence of false positives, which are not likely to be detected on different branches of a same gene family, should be reduced. In addition, the gene set enrichment approach reduces the contribution of individual genes and rather favors functional categories including many good scoring genes. We observed that most of the top significant GO categories displayed a global shift towards large scores at the positive selection test, and were not called significant because of a single gene with an extreme high score (Figure S7).

Different post-filtering steps of the data indicated that the functional enrichment patterns were not driven by methodological biases or false positives. The results of the enrichment test were similarly consistent after filtering out i) all foreground terminal branches ("leaves"; Table S20); ii) all branches which displayed a d_S greater than 1 (Table S21); iii) all positives where the identification of positively selected residues in protein alignments failed (Table S22); iv) all branches where the alternative or the fixed model displayed convergence issues (Table S23); and v) all branches where the increase in G+C content at third codon positions was higher than 10% (Table S24). They were also consistent when the enrichment test was based on results from the dataset reprocessed with a different aligner (PAGAN) (Loytynoja, Vilella, Goldman 2012) and a different filtering method (GUIDANCE)(Penn et al. 2010; Privman, Penn, Pupko 2012)(Table S25), or subjected to a different test for positive selection, the site test (see above). Thus the functional insights from our scan for positive selection seemed quite robust.

Overall, our methodological choices may hamper the detection of a significant portion of positive selection events (e.g., species-specific events), but we feel that such high standard quality control steps should be applied systematically previous to large-scale scans for positive selection as they greatly reduce the rate of

false positives, which is otherwise a major concern (Markova-Raina, Petrov 2011). The benefit of alignment filtering has been estimated to exceed the loss of power due to the removal of some of the true positively selected sites, based on simulations (Jordan, Goldman 2012; Privman, Penn, Pupko 2012). Even after multiple quality checks, false positives may not be totally eliminated, which is why we favored a post-processing of the results that should not be very sensitive to the influence of such false positives.

Gene set enrichment test with KEGG pathways

To verify that the results of the gene set enrichment results were not strongly dependent on the annotation of Gene Ontology categories, we performed another enrichment test based on the annotation of KEGG pathways for *D. melanogaster* genes (Table S26). There was a significant enrichment for positively selected genes among genes annotated with the general term "metabolic pathways". In particular, fatty acid, amino-acid, nucleotide, sugar, and the oxidative phosphorylation metabolic pathways were enriched. These results are consistent with the enrichment of Gene Ontology categories related to proteolysis, metabolism and mitochondrial activity (Table 3, Table S6, Table S25). In addition there was a significant enrichment for pathways related to translation ("ribosome" and "ribosome biogenesis in eukaryotes") which were also previously observed with the Gene Ontology. Only the enrichment for the "RNA polymerase" pathway, composed of the constituents of the RNA polymerase complex, was not previously reported with the test based on Gene Ontology, but was in fact very close to the significance cutoff (e.g., FDR=22% for GO category "RNA polymerase activity"; Table S6).

Characterization of functional biases in the datasets

Gene families were filtered based on their topology and on the quality of their sequences (Materials and Methods). We sought to characterize the biases of the subsets of gene families used in this study compared to all annotated gene families available for the set of species analyzed. The single-copy orthologs dataset in ants shows an enrichment of genes linked to translation and GTPase activity, while genes

with receptor (e.g., neuropeptide, ionotropic glutamate, olfactory), catalytic (e.g., oxidoreductase, lipase, transaminase, peptidase), binding (e.g., vitamin, metal ion, DNA) or structural molecule (e.g., structural constituent of chitin-based cuticle) activity are depleted (Table S8). Some of the latter are of particular interest to ant biology but their depletion leads to a decreased power to analyze functional biases among positively selected genes.

The extensive dataset, including gene families that experienced gene duplication, gathered all OrthoDB gene families that passed our basic quality filters. But essentially the same functional categories were depleted, with the addition of taste receptor activity, defense response and spermatid nucleus differentiation (Table S10). These functional categories probably gather genes whose annotation is still incomplete in the analyzed genomes and require manual annotation. The functional categories that gained representation in this dataset are linked to transcriptional regulation: DNA binding, transcription factor (e.g., general RNA polymerase II transcription factor activity is enriched) and RNA binding proteins involved in mRNA splicing.

Of note, there were substantial differences with the functional biases found in datasets of single-copy orthologs from the 12 *Drosophila* species (Drosophila 12 Genomes Consortium 2007) or from 10 bee species (Woodard et al. 2011), that were processed through the same quality filtering pipeline (Table S7 and S11; Materials and Methods). The *Drosophila* dataset was enriched in odorant and pheromone binding and in genes involved in mitochondrial processes (e.g., NADH dehydrogenase and oxidoreductase activity, oxidation-reduction process, mitochondrial electron transport, mitochondrial DNA replication), while it was depleted in genes involved in chromatin organization, reproduction, brain development and morphogenesis (eye, wing). The bee dataset was enriched in protein folding activity, regulation of splicing and translation, and mitochondrial processes as well. Quite similar to ants, it was depleted in receptor activity (odorant, neurotransmitter, neuropeptide steroid hormone and ionotropic glutamate), but also in immune functions, transcription factor activity and morphogenesis (embryo, wing, leg, nervous system, tracheal system, ventral cord, midgut).

Comparison with other lineages

We compared our ant genomes dataset with the largest lineage-specific genomic datasets available for other insect groups. The first dataset gathers twelve genomes of *Drosophila* species (Diptera)(Drosophila 12 Genomes Consortium 2007). The second dataset gathers gene models reconstructed from expressed sequence tags (ESTs) from nine bee species and the genome of the honey bee *Apis mellifera* (Woodard et al. 2011). This dataset is of particular interest because species of the bee lineage acquired sociality several times independently. Although positive selection scans were previously performed for these datasets, the differences in methodology hinder direct comparisons with our results. For example the fly dataset was analyzed with the site test of Codeml and the bee dataset was analyzed with the branch test of Codeml. Thus we applied the exact same alignment filtering methodology to these data as to the ant data, the same branch-site positive selection test, and the same gene set enrichment test on Gene Ontology functional categories on these two datasets. Differences in topology of the phylogenetic tree of the species cannot be directly controlled for and can be responsible for differences in power of the positive selection test. Similarly, difference in selective pressure at synonymous sites and mutational biases may vary between lineages. Yet, the common methodological framework maximizes chances that differences in the results from these scans can be attributed to biological differences with ants.

In the twelve *Drosophila* dataset, at FDR 10%, 1,365 gene families out of 3,749 (36%) displayed some significant signal of positive selection in at least one branch of the tree, with a maximum of 9.5% positives in the branch leading to the Subgenus *Sophophora*, and a minimum of 0.30% in the branch leading to *D. yakuba* and *D. erecta* (Table S3). In the bee dataset, 461 gene families out of 2,256 displayed episodes of positive selection (20%), with a maximum of 5.8% positives in the branch leading to *Euglossa cordata*, and a minimum of 0.31% in the branch leading to *Apis mellifera* (Table S2). Reassuringly, the numbers of positives were on the same orders of magnitude in the ant, fly and bee datasets .

For the gene set enrichment test, similarly to ants, we considered test scores on all branches of the lineage. The long basal branches leading to *Megachile rotundata* and *Exoneura robusta* where excluded from the bee dataset. In flies, there was an enrichment of positive selection for functions related to chitin binding, metabolism, immune response, proteolysis, receptor activity (e.g., olfactory receptor activity), response to stimulus, localization in the cell, meiosis, membrane, nuclear envelope, extracellular region, chromosome organization, spermatogenesis, regulation of cell cycle, negative regulation of gene expression and wing disc development (Table S4). Mitochondria-related terms were largely absent, with the exception of oxidoreductase activity (but not ranked on top of the list; FDR = 9%). In the bee lineage, positively selected genes belonged to functional categories such as GTPase activity, receptor activity, metabolism, response to hormone stimulus, localization in the cell and proteolysis (Table S5). There was no significant term associated to mitochondria. This is not due to a power issue for the gene set enrichment test since genes with mitochondrial functions appear to be enriched in both the fly and bee datasets (Table S7 and S11). Aside from mitochondria, and more particularly in the bee lineage, the functional categories identified are broadly similar to results in ants. It is difficult to imagine how differences in species tree topology and substitutions patterns – the only differences that could have an impact on the performance of the branch-site test between the three scans – would affect exclusively mitochondrial functions.

Examples of Codeml control files for the branch-site test

Alternative hypothesis

```
     seqfile = 15.phy     * sequence data file name
    treefile = 15.01.nwk  * tree structure file name
     outfile = 15.01.mlc  * main result file name

       noisy = 9   * 0,1,2,3,9: how much rubbish on the screen
     verbose = 1   * 1: detailed output, 0: concise output
     runmode = 0   * 0: user tree;  1: semi-automatic;  2: automatic
                   * 3: StepwiseAddition; (4,5):PerturbationNNI; -2: pairwise

     seqtype = 1   * 1:codons; 2:AAs; 3:codons-->AAs
   CodonFreq = 2   * 0:1/61 each, 1:F1X4, 2:F3X4, 3:codon table
       ndata = 1   * specifies the number of separate data sets in the file
       clock = 0   * 0: no clock, unrooted tree, 1: clock, rooted tree
      aaDist = 0   * 0:equal, +:geometric; -:linear, {1-5:G1974,Miyata,c,p,v}
       model = 2   * models for codons:
                   * 0:one, 1:b, 2:2 or more dN/dS ratios for branches
     NSsites = 2   * 0:one w; 1:NearlyNeutral; 2:PositiveSelection; 3:discrete;
                   * 4:freqs; 5:gamma;6:2gamma;7:beta;8:beta&w;9:beta&gamma;10:3normal
       icode = 0   * 0:standard genetic code; 1:mammalian mt; 2-10:see below
       Mgene = 0   * 0:rates, 1:separate; 2:pi, 3:kappa, 4:all

   fix_kappa = 0   * 1: kappa fixed, 0: kappa to be estimated
       kappa = 2   * initial or fixed kappa
   fix_omega = 0   * 1: omega or omega_1 fixed, 0: estimate
       omega = 1   * initial or fixed omega, for codons or codon-based AAs

       getSE = 0   * 0: don't want them, 1: want S.E.s of estimates
RateAncestor = 0   * (0,1,2): rates (alpha>0) or ancestral states (1 or 2)
  Small_Diff = .5e-6
   cleandata = 1   * remove sites with ambiguity data (1:yes, 0:no)?
 fix_blength = 0   * 0: ignore, -1: random, 1: initial, 2: fixed
      method = 0   * 0: simultaneous; 1: one branch at a time
```

Null model

```
     seqfile = 15.phy     * sequence data file name
    treefile = 15.01.nwk  * tree structure file name
     outfile = 15.01.fx.mlc  * main result file name

       noisy = 9   * 0,1,2,3,9: how much rubbish on the screen
     verbose = 1   * 1: detailed output, 0: concise output
     runmode = 0   * 0: user tree;  1: semi-automatic;  2: automatic
                   * 3: StepwiseAddition; (4,5):PerturbationNNI; -2: pairwise

     seqtype = 1   * 1:codons; 2:AAs; 3:codons-->AAs
   CodonFreq = 2   * 0:1/61 each, 1:F1X4, 2:F3X4, 3:codon table
       ndata = 1   * specifies the number of separate data sets in the file
       clock = 0   * 0: no clock, unrooted tree, 1: clock, rooted tree
      aaDist = 0   * 0:equal, +:geometric; -:linear, {1-5:G1974,Miyata,c,p,v}
       model = 2   * models for codons:
                   * 0:one, 1:b, 2:2 or more dN/dS ratios for branches
     NSsites = 2   * 0:one w; 1:NearlyNeutral; 2:PositiveSelection; 3:discrete;
                   * 4:freqs; 5:gamma;6:2gamma;7:beta;8:beta&w;9:beta&gamma;10:3normal
       icode = 0   * 0:standard genetic code; 1:mammalian mt; 2-10:see below
       Mgene = 0   * 0:rates, 1:separate; 2:pi, 3:kappa, 4:all

   fix_kappa = 0   * 1: kappa fixed, 0: kappa to be estimated
       kappa = 2   * initial or fixed kappa
   fix_omega = 1   * 1: omega or omega_1 fixed, 0: estimate
       omega = 1   * initial or fixed omega, for codons or codon-based AAs
```

```
       getSE = 0   * 0: don't want them, 1: want S.E.s of estimates
RateAncestor = 0   * (0,1,2): rates (alpha>0) or ancestral states (1 or 2)
  Small_Diff = .5e-6
   cleandata = 1   * remove sites with ambiguity data (1:yes, 0:no)?
 fix_blength = 0   * 0: ignore, -1: random, 1: initial, 2: fixed
      method = 0   * 0: simultaneous; 1: one branch at a time
```

Examples of Codeml control files for the site test

Alternative hypothesis (M8)

```
      seqfile = cds.GUIDANCE.pagan.nnn.0.8.fa
     treefile = cds.GUIDANCE.pagan.nnn.0.8.raxml
      outfile = M8.out

        noisy = 9  * 0,1,2,3,9: how much rubbish on the screen
      verbose = 2  * 0: concise; 1: detailed, 2: too much
      runmode = 0  * 0: user tree;  1: semi-automatic;  2: automatic
                   * 3: StepwiseAddition; (4,5):PerturbationNNI; -2: pairwise
      seqtype = 1  * 1:codons; 2:AAs; 3:codons-->AAs
    CodonFreq = 2  * 0:1/61 each, 1:F1X4, 2:F3X4, 3:codon table
*        ndata = 1
        clock = 0  * 0:no clock, 1:clock; 2:local clock; 3:CombinedAnalysis
       aaDist = 0  * 0:equal, +:geometric; -:linear, 1-6:G1974,Miyata,c,p,v,a
   aaRatefile = /home/talpu/rubi/paml44/dat/wag.dat

        model = 0
                   * models for codons:
                       * 0:one, 1:b, 2:2 or more dN/dS ratios for branches
                   * models for AAs or codon-translated AAs:
                       * 0:poisson, 1:proportional, 2:Empirical, 3:Empirical+F
                       * 6:FromCodon, 7:AAClasses, 8:REVaa_0, 9:REVaa(nr=189)

      NSsites = 8  * 0:one w;1:neutral;2:selection; 3:discrete;4:freqs;
                   * 5:gamma;6:2gamma;7:beta;8:beta&w;9:beta&gamma;
                   * 10:beta&gamma+1; 11:beta&normal>1; 12:0&2normal>1;
                   * 13:3normal>0

        icode = 0  * 0:universal code; 1:mammalian mt; 2-10:see below
        Mgene = 0
                   * codon: 0:rates, 1:separate; 2:diff pi, 3:diff kapa, 4:all diff
                   * AA: 0:rates, 1:separate

    fix_kappa = 0  * 1: kappa fixed, 0: kappa to be estimated
        kappa = 2  * initial or fixed kappa
    fix_omega = 0  * 1: omega or omega_1 fixed, 0: estimate
        omega = 1 * initial or fixed omega, for codons or codon-based AAs

*    fix_alpha = 1  * 0: estimate gamma shape parameter; 1: fix it at alpha
*        alpha = 0. * initial or fixed alpha, 0:infinity (constant rate)
*       Malpha = 0  * different alphas for genes
        ncatG = 8  * # of categories in dG of NSsites models

        getSE = 1  * 0: don't want them, 1: want S.E.s of estimates
 RateAncestor = 0  * (0,1,2): rates (alpha>0) or ancestral states (1 or 2)

   Small_Diff = .5e-6
    cleandata = 0  * remove sites with ambiguity data (1:yes, 0:no)?
  fix_blength = 1  * 0: ignore, -1: random, 1: initial, 2: fixed
       method = 0  * Optimization method 0: simultaneous; 1: one branch a time
```

Null model (M8a)

```
      seqfile = cds.GUIDANCE.pagan.nnn.0.8.fa
     treefile = cds.GUIDANCE.pagan.nnn.0.8.raxml
      outfile = M8a.out
```

```
        noisy = 9  * 0,1,2,3,9: how much rubbish on the screen
      verbose = 2  * 0: concise; 1: detailed, 2: too much
      runmode = 0  * 0: user tree;  1: semi-automatic;  2: automatic
                   * 3: StepwiseAddition; (4,5):PerturbationNNI; -2: pairwise

      seqtype = 1  * 1:codons; 2:AAs; 3:codons-->AAs
    CodonFreq = 2  * 0:1/61 each, 1:F1X4, 2:F3X4, 3:codon table
*        ndata = 1
        clock = 0  * 0:no clock, 1:clock; 2:local clock; 3:CombinedAnalysis
       aaDist = 0  * 0:equal, +:geometric; -:linear, 1-6:G1974,Miyata,c,p,v,a
   aaRatefile = /home/talpu/rubi/paml44/dat/wag.dat

        model = 0
                   * models for codons:
                       * 0:one, 1:b, 2:2 or more dN/dS ratios for branches
                   * models for AAs or codon-translated AAs:
                       * 0:poisson, 1:proportional, 2:Empirical, 3:Empirical+F
                       * 6:FromCodon, 7:AAClasses, 8:REVaa_0, 9:REVaa(nr=189)

      NSsites = 8  * 0:one w;1:neutral;2:selection; 3:discrete;4:freqs;
                   * 5:gamma;6:2gamma;7:beta;8:beta&w;9:beta&gamma;
                   * 10:beta&gamma+1; 11:beta&normal>1; 12:0&2normal>1;
                   * 13:3normal>0

        icode = 0  * 0:universal code; 1:mammalian mt; 2-10:see below
        Mgene = 0
                   * codon: 0:rates, 1:separate; 2:diff pi, 3:diff kapa, 4:all diff
                   * AA: 0:rates, 1:separate

    fix_kappa = 0  * 1: kappa fixed, 0: kappa to be estimated
        kappa = 2  * initial or fixed kappa
    fix_omega = 1  * 1: omega or omega_1 fixed, 0: estimate
        omega = 1 * initial or fixed omega, for codons or codon-based AAs

*    fix_alpha = 1  * 0: estimate gamma shape parameter; 1: fix it at alpha
*        alpha = 0. * initial or fixed alpha, 0:infinity (constant rate)
*       Malpha = 0  * different alphas for genes
        ncatG = 8  * # of categories in dG of NSsites models

        getSE = 1  * 0: don't want them, 1: want S.E.s of estimates
 RateAncestor = 0  * (0,1,2): rates (alpha>0) or ancestral states (1 or 2)

   Small_Diff = .5e-6
    cleandata = 0  * remove sites with ambiguity data (1:yes, 0:no)?
  fix_blength = 1  * 0: ignore, -1: random, 1: initial, 2: fixed
       method = 0  * Optimization method 0: simultaneous; 1: one branch a time
```

Newick files for 16 olfactory receptors subtrees

"#" characters indicate the IDs of tested branches, as reported in Table S18.

Clade 1:

```
(LhOr214#52:0.12700270391109774737,PbOr155#0:0.15115695719430483068,((PbOr162#40:0.127670
95981004664140,(PbOr156FIX#41:0.06912065723405018403,(PbOr160FIX#47:0.0543540491807749798
2,(PbOr159FIX#45:0.04849218372794571880,(PbOr158FIX#42:0.02906122433329262275,PbOr157#43:
0.03376281725807433326)#44:0.03942050956533193345)#46:0.04646045906677091081)#48:0.032683
18554372971058)#49:0.04611026577420087452)#50:0.20155627979239407188,(NvOr261#38:0.331896
33324758538446,((((NvOr256#30:0.07472817364594770695,(NvOr258#32:0.05039805107455570116,N
vOr257#31:0.05050608904167195462)#33:0.07834442018658442641)#34:0.16938432354919935596,(N
vOr255#28:0.18584305712397086729,NvOr260#27:0.25135472314584683673)#29:0.0487927341428094
9073)#35:0.05442767623539263022,((NvOr245#8:0.11949917114384320249,((NvOr262#10:0.0413044
8977170079333,NvOr263#9:0.04937130563266019928)#11:0.07173047213223919527,(NvOr241#13:0.0
3649830016750404277,NvOr243#12:0.08993844906210475554)#14:0.03713426930879297483)#15:0.05
905111656602962666)#16:0.07312961651287799847,((NvOr252#18:0.05010656346318344972,NvOr251
#17:0.05410011513330496846)#19:0.21025708466762274562,(NvOr253#20:0.15018635430508897599,
(NvOr250#21:0.03185269629544653508,NvOr247#22:0.07211623334040800914)#23:0.13973691861200
```

876474)#24:0.05363800920876846817)#25:0.06757557137934989966)#26:0.16156367801895024638)#
36:0.04019419297325978885,((NvOr249#4:0.16438483878311427322,NvOr246#5:0.1750449589666619
7319)#6:0.06183094703029002465,(NvOr242#1:0.25114299996151995265,NvOr248#2:0.296449827148
14260838)#3:0.09209660106446855565)#7:0.18014489351874354850)#37:0.11547797001294089758)#
39:0.20359534912102003612)#51:0.14421525008022353931)#53;

Clade 2:

(PbOr176#73:0.06082873108860709088,LhOr215#74:0.08353345534761674740,(((LhOr218#39:0.0050
859522971383513,LhOr219FIX#40:0.01306812986974399855)#41:0.20309753047680537130,((((PbOr
192#43:0.08396852117914523073,PbOr184NEW#42:0.08825892051780954983)#44:0.0501940963470112
1892,(LhOr220#46:0.05553828515331037108,PbOr182NEW#45:0.05937380831970199419)#47:0.087141
72575459538939)#48:0.02882557447453374527,(PbOr177#50:0.08066248512340329357,PbOr185#49:0
.09420379603840116356)#51:0.11039959084735397055)#52:0.08017725547537361830,((PbOr189#53:
0.07376054307363721918,PbOr187#54:0.09381812878975828718)#55:0.11704343743263055655,((PbO
r180#66:0.07438442813359358308,LhOr217FIX#65:0.09832388052481165486)#67:0.113337604740720
96525,((PbOr188FIX#57:0.07319211841743798741,LhOr216#56:0.07929826180456862883)#58:0.0768
1711963909131080,(PbOr179#59:0.15116957436341549315,(PbOr190FIX#61:0.09502974931755860111
,PbOr178#60:0.13593561026870532027)#62:0.05975825668385024991)#63:0.04295060459312043805)
#64:0.03585467346065920186)#68:0.03290278308586715161)#69:0.04213635842695823142)#70:0.02
724460764805466950)#71:0.04972358634563252278,(NvOr44#0:0.20983718073005075411,((NvOr45#1
:0.24374515880998551265,((PbOr194JF#7:0.10132752242257116182,((PbOr195JF#4:0.073185529271
35921204,LhOr222#3:0.09151725482589040395)#5:0.02551164126895187970,PbOr193JOI#2:0.133269
93861774322214)#6:0.06923954708520733969)#8:0.05670835241989650999,(PbOr181#12:0.17233206
426544503409,(PbOr196JF#10:0.06728442567136626307,LhOr223JOI#9:0.07632338427646809254)#11
:0.14171167374924883986)#13:0.06989996587889162061)#14:0.06403208467685669869)#15:0.04267
581126975338729,((NvOr43#19:0.17630549199790204740,(NvOr46#17:0.18846345356926758052,PbOr
191FIX#16:0.21852116812660760115)#18:0.09838471065440075602)#20:0.10050417797038178891,((
NvOr48#32:0.20520479115547776705,NvOr47#33:0.23529211380457948355)#34:0.06234813422945846
562,((NvOr59#21:0.17979942748144203679,NvOr58#22:0.20537659525162291208)#23:0.08142301811
164054381,(NvOr56#24:0.23087166119626123528,(NvOr53#28:0.14582347574470266860,(NvOr50#26:
0.17407287249632374948,NvOr51#25:0.19668754689129891444)#27:0.05245509519470525212)#29:0.
06182327394637425477)#30:0.04324976757348040352)#31:0.05404031374042227165)#35:0.11063913
796319144178)#36:0.06659586664272680590)#37:0.08250691010857801044)#38:0.0545756770212654
1603)#72:0.13964315019539036422)#75;

Clade 3:

(((LhOr47FIX#2:0.14825294768755742369,PbOr163#1:0.15484356746550523298)#3:0.0600684727292
9938253,PbOr166#0:0.28579803732196529253)#4:0.09666298326824580844,LhOr59#38:0.4217260862
0646406807,(((PbOr168#8:0.16760099190192406793,(LhOr50#6:0.01912291658990686663,LhOr51#5:
0.04623685283333146823)#7:0.14352593428938073550)#9:0.11706087756188153504,((LhOr55#13:0.
13871445744291385771,(LhOr52#14:0.15731064777396269738,PbOr170#15:0.17681619961022698728)
#16:0.03308287452757098512)#17:0.08394046750557647429,(LhOr54#10:0.11112369129639920740,L
hOr56#11:0.18554622372035137201)#12:0.13125253993846636158)#18:0.04522372944504170211)#19
:0.02845752858792141224,((PbOr172#20:0.14523439070950164287,(LhOr57#22:0.1382224018345351
8569,LhOr58#21:0.14275377695965757452)#23:0.04138473458732910837)#24:0.079498667415364931
98,((PbOr173#26:0.18328208402641280039,PbOr175#25:0.22942283637932850415)#27:0.0650101539
0752930279,((LhOr49#31:0.12410098473386869944,PbOr167#32:0.15034893536074842024)#33:0.041
85656246491760435,(LhOr48#29:0.14575579529538179591,PbOr164FIX#28:0.18741919016634589479)
#30:0.04616045473236166791)#34:0.10822836285965163616)#35:0.04171216768762579796)#36:0.03
249126373429089831)#37:0.04693668565656346908)#39;

Clade 4:

(LhOr224#194:0.06504435792040369479,PbOr197#193:0.08348903213533781509,((((PbOr227#0:0.06
176522391653117838,(LhOr227#2:0.02200234638084715838,LhOr228#1:0.04724448386920492710)#3:
0.04933393142581436741)#4:0.07998914245664295875,((LhOr229#11:0.04676674479915721161,PbOr
229#10:0.07388743043047751868)#12:0.06284001202834139932,(LhOr230#8:0.1033557767361987928
0,(PbOr230#5:0.07511824212747636453,LhOr231#6:0.09112009180771077355)#7:0.043353440178097
14123)#9:0.06507274472893065376)#13:0.03903418690337849550)#14:0.09366516484213024962,(((
NvOr126#15:0.05589359230633769021,NvOr124#16:0.07604144192067412156)#17:0.047364039388775
26559,NvOr128#18:0.13384899451331291886)#19:0.13331641230614832416,((NvOr119#20:0.0444232
2241162317831,(NvOr117#22:0.04605681412635846683,NvOr118#21:0.05045349731899097939)#23:0.
02699852754059564250)#24:0.18898956931021917982,(NvOr125#26:0.18233343229472204206,NvOr12
2#25:0.20428820857952811885)#27:0.07152642358899721775)#28:0.02595831089852023571)#29:0.0
4611936372324701439)#30:0.10842611891270696745,((((NvOr107#34:0.08932820614844806661,(NvO
r110#31:0.06700104287603399211,NvOr111#32:0.08957835361537108687)#33:0.073289835542808573
49)#35:0.14770157120687424368,((NvOr115#37:0.14610190769251085730,NvOr114#36:0.1530407003
9950565252)#38:0.04953351894107758946,(NvOr106#40:0.14477996999197065287,NvOr113#39:0.230

25840044462658751)#41:0.03996184879526618050)#42:0.12922378682444354836)#43:0.09057858589
996808929,(NvOr98#55:0.20515600813616502451,(NvOr105#53:0.21531200668660840924,(NvOr103#4
4:0.19783488303960186649,(NvOr99#45:0.22934881716895622050,(NvOr100#49:0.1107646489724804
6448,(NvOr101#47:0.11609804066249530818,NvOr102#46:0.13329996092290880005)#48:0.042743476
43710470906)#50:0.10890853287333576715)#51:0.05764146843752000759)#52:0.0634549714666404 8
904)#54:0.12119801847062493250)#56:0.07158943013685371837)#57:0.05065487739115683702,(((L
hOr226#178:0.17173552585749210508,(LhOr225#179:0.08072203317854952098,((PbOr200#180:0.012
3988453862984553 8,PbOr201#181:0.01729820481465962900)#182:0.02873908024839657779,(PbOr199
FIX#184:0.05133291107751279669,PbOr198FIX#183:0.05746493662568850219)#185:0.0554710823071
7896077)#186:0.03499219007062884940)#187:0.09302635729937655529)#188:0.10016611487049059 6
81,NvOr80#177:0.34513156863301325261)#189:0.04007187567712019310,(((NvOr93#159:0.13838922
397062994318,NvOr94#160:0.25105031492866136666)#161:0.08730431035082647506,((NvOr92#162:0
.21111631019008858523,NvOr96#163:0.25412796264462572671)#164:0.07893165543625271785,(NvOr
85#172:0.26004568330752331207,(NvOr87#165:0.06937547196974769292,(NvOr86#166:0.0896171618
4643699068,(NvOr89#168:0.09128589151461544149,NvOr88#167:0.11637507572930734512)#169:0.03
68488684902636490 2)#170:0.03759933528303834038)#171:0.16661684623894640334)#173:0.0936092
694648853196 3)#174:0.03456452198989460461)#175:0.06001594860682312599,((NvOr82#131:0.2353
151351152511505 7,(((LhOr138#132:0.07617950621418133339,LhOr137#133:0.08173779416394665043
)#134:0.02630187408907139548,(PbOr202FIX#135:0.09255335379798800000,LhOr139#136:0.1043611
5056603871565)#137:0.04170959979407941332)#138:0.06615103520112568247,(LhOr144#139:0.0859
709073537214957 3,(LhOr143#153:0.05383791593079666898,((PbOr207#140:0.07908855815766097086
,LhOr145#141:0.08047765722439555180)#142:0.01432419604896608377,((LhOr142#143:0.022254056
86454412155,LhOr141#144:0.02612123451726530710)#145:0.05835728971430727274,(LhOr140FIX#14
6:0.06023088414085454867,(PbOr203#147:0.05048176564343271472,PbOr206FIX#148:0.07293497926
021914879)#149:0.02445997336843486192)#150:0.01800266797363784740)#151:0.0218456176052402
4454)#152:0.01919369160337373870)#154:0.02293161076719913913)#155:0.08206439832863024475)
#156:0.13007903628812014785)#157:0.03629492365495076006,(NvOr81#58:0.26116659387283153171
,(((LhOr129#126:0.15463431011578696039,(LhOr130#117:0.09259255782748161689,(LhOr131#118:0
.07338436863986312619,((PbOr219#120:0.00657755211625470765,PbOr220#119:0.009708720254763 6
5690)#121:0.03126149524840589333,PbOr221#122:0.04618442653090105987)#123:0.0501134038938 1
239296)#124:0.04710643812301686106)#125:0.05646309651803915058)#127:0.0299696386226432978
7,(PbOr218#98:0.13817529405412767862,(((PbOr223JOI#102:0.09554340064719422931,LhOr133#103
:0.09607330263517104008)#104:0.06773160129828507958,((PbOr225#110:0.05840631065714693010,
LhOr136#111:0.06129550243431721585)#112:0.02863205998608612038,(PbOr224JOI#105:0.05967930
446623592039,(LhOr134FIX#106:0.03882030489971165926,LhOr135#107:0.05245443603005721378)#1
08:0.02734100982652727663)#109:0.02221943921972408670)#113:0.06229415240300216483)#114:0.
04425715165649175831,(PbOr222FIX#100:0.06781266718986005138,LhOr132#99:0.0967153897948639
3739)#101:0.12874722722204420822)#115:0.02930940349042825133)#116:0.03016716955933224781)
#128:0.02784808303268420174,((LhOr127#59:0.10112494322657009094,(LhOr128#61:0.06766822651
738080285,PbOr217FIX#60:0.08930768486368823034)#62:0.06053751376895628172)#63:0.029995758
53513243573,((PbOr215#94:0.05952538249328418601,LhOr126#93:0.06817009604892103336)#95:0.0
805143987688307499 8,((LhOr123FIX#85:0.10941278305232279844,((LhOr124#87:0.00264956397106 8
87306,LhOr125#86:0.00392835989778709052)#88:0.06142321146866561860,PbOr214#89:0.074580948
14498580916)#90:0.05655613561670068401)#91:0.05219033802780467801,((PbOr213#81:0.05631993
23143697000 4,LhOr122#82:0.06450614786253108801)#83:0.06158604113478619024,(((LhOr120FIX#6
5:0.05896843084347736658,PbOr211JF#64:0.07061465707509259615)#66:0.02880401579988308564,(
LhOr121#67:0.06197455293106976515,PbOr212#68:0.08059420917722724675)#69:0.029553833821262
20912)#70:0.02920296751061459903,(LhOr117#78:0.10785050805581858357,(LhOr119#76:0.0738046
8065395255628,(LhOr118#74:0.05954272437315043937,(PbOr210#71:0.03898825948681169246,PbOr2
09#72:0.04025791166585995784)#73:0.05318882247522752765)#75:0.02894409437281996941)#77:0.
04348617686233215152)#79:0.02627827371297620021)#80:0.01797369849325510666)#84:0.02038427
057785799010)#92:0.03912442310666421064)#96:0.02786144984910120595)#97:0.0293231894675958
9554)#129:0.12825981947677952966)#130:0.06444625853748782440)#158:0.04837069543325687310)
#176:0.02960603465005828480)#190:0.06427556076885039549)#191:0.11715824898224512507)#192:
0.31032493451553094799)#195;

Clade 5:

(LhOr249#20:0.11567698648139186635,(PbOr247#0:0.13613441893244399017,(PbOr250#2:0.1384167
6826479470952,PbOr248FIX#1:0.22504147022611128981)#3:0.05170688739146375029)#4:0.06267954
107689907572,(LhOr250#5:0.15260238988977392616,((PbOr255#6:0.14427136058744652325,(PbOr25
4#7:0.10596592998757430049,(PbOr252#9:0.02964662589210982982,PbOr253#8:0.0302365662747951
6176)#10:0.08071848342509982399)#11:0.07558754439449749674)#12:0.04707406066343369777,(Pb
Or251#13:0.18207115617410660202,(PbOr259#15:0.01342003393894021339,PbOr258FIX#14:0.021438
45300876345492)#16:0.17807297464121768504)#17:0.04651479841059385362)#18:0.07434689003337
921487)#19:0.36097074054056921266)#21;

Clade 6:

```
(LhOr232#16:0.11833550742016626933,(PbOr233#1:0.06766645099729103052,PbOr234#0:0.12669581
414876343595)#2:0.13923645879975890538,(((LhOr234#6:0.08271829219557245982,LhOr235#7:0.08
71130687554488025 7)#8:0.08546456401918384183,(PbOr240#4:0.16224982501208068397,PbOr237#3:
0.16991316763588712457)#5:0.08139165401930825716)#9:0.08259964582320121484,((LhOr238#11:0
.10235310175682220402,PbOr242#12:0.11811708940859366734)#13:0.05670355409185014878,PbOr24
1#10:0.23964131025281165677)#14:0.09628328062232598128)#15:0.05969708117238837347)#17;
```

Clade 7:

```
(LhOr252#46:0.09688591177139660449,(PbOr260FIX#41:0.10329776120361008895,(PbOr262#43:0.01
30152248213839510 2,PbOr261#42:0.02030291376702532055)#44:0.11607996400798416270)#45:0.070
0620717621074362 6,((LhOr253#1:0.11031350479778091744,PbOr263#0:0.13967675180880764763)#2:
0.21505399416570683546,(((LhOr337#3:0.07950616143974434391,PbOr269#4:0.08453503880021727 4
24)#5:0.13487450679949991583,(PbOr268#7:0.08685297892073672033,LhOr263#6:0.08991687232595
861612)#8:0.18168917448194313313)#9:0.14013391588614240724,((PbOr267#10:0.087386717834761
44317,LhOr262#11:0.08902269464669325394)#12:0.31697304030811540576,((LhOr254FIX#13:0.0863
3555337985482359,(LhOr256#14:0.09335724476444627606,PbOr264#15:0.12012054948630616902)#16
:0.04167234786928292883)#17:0.23864328947706012207,(PbOr266#18:0.23793243351172899125,(Lh
Or257#19:0.13663783793552455870,(((LhOr259#20:0.04371035943999277557,LhOr261#21:0.0568462
334136563968 2)#22:0.05428732855120772804,LhOr255#23:0.13905622541572784656)#24:0.05417510
570998938096,((LhOr244#28:0.12923892990161073291,(LhOr242#30:0.10047284838189202150,LhOr2
43#29:0.10571426844887030216)#31:0.04005036931491708779)#32:0.01630986891898068836,(LhOr2
60FIX#25:0.15048057649794957524,LhOr241#26:0.15485882046963569958)#27:0.03048673398894139
602)#33:0.05879451328688677092)#34:0.09416933725900324126)#35:0.06413693161577253832)#36:
0.24218979410413338238)#37:0.08906417945140271408)#38:0.05129636866962993674)#39:0.129826
46880680215329)#40:0.24335335713919573064)#47;
```

Clade 8:

```
(LhOr240#0:0.02659375220041562335,LhOr239#50:0.03797266272919302965,(PbOr244#48:0.1459820
1985403902459,((LhOr245#1:0.25494240555791736202,((PbOr382#3:0.00000093905322139818,PbOr1
50#4:0.00000093905322139818)#5:0.14050117565847597412,LhOr367#2:0.18525005025552168658)#6
:0.15194325809032838137)#7:0.06438092334391085081,((LhOr246#8:0.13820783637317637926,(PbO
r245FIX#9:0.01896935363422937865,PbOr246#10:0.02081636438420580645)#11:0.1248612003697734
3961)#12:0.14309015851042611311,(((LhOr355#19:0.06046077939710672866,(LhOr357#16:0.039627
93750964297562,LhOr358#17:0.04929105040360693551)#18:0.06991349285795393775)#20:0.0345234
9443888502734,(LhOr356#13:0.06815799862762034200,LhOr360FIX#14:0.10100239059699699096)#15
:0.05621657844326546222)#21:0.10382267040952466997,(PbOr372#43:0.19391114791672720008,(((
PbOr373FIX#22:0.09364892526891381574,LhOr361#23:0.12791185056684262200)#24:0.057013009623
36082467,(PbOr374#25:0.07508352365020541652,((LhOr364FIX#27:0.00031878436060032766,LhOr36
2FIX#26:0.01829359455369910376)#28:0.05351848543071109632,LhOr363#29:0.091046367824473992
13)#30:0.05459504079448435077)#31:0.10868924012011385583)#32:0.02828069977601530699,((LhO
r365#33:0.11310538629193366000,LhOr366#34:0.11898173668373671075)#35:0.032552039125915734
06,(PbOr381FIX#39:0.11029154115902831124,(PbOr380#37:0.11561917985044264934,PbOr377#36:0.
16327925040951529745)#38:0.03478559067589177822)#40:0.03058909707610408396)#41:0.06288362
291539412174)#42:0.04908083236129496402)#44:0.05006665087162787131)#45:0.231050386170117 4
4440)#46:0.05433629773047895378)#47:0.41271540793973204853)#49:0.15800151646562046737)#51
;
```

Clade 9:

```
(LhOr349#38:0.10653689323086852625,PbOr400#37:0.12125869977855327497,(PbOr274#35:0.225768
61578153556431,((PbOr280#0:0.17957430472531507593,(PbOr277FIX#2:0.08958783734220689132,Pb
Or275#1:0.10626137563587727264)#3:0.11025367672366340188)#4:0.13232702713950048046,(((PbO
r271#5:0.12589958044388102620,PbOr273#6:0.15242820168087825583)#7:0.03691392816308682739,
(LhOr348#8:0.14433441609673261508,(LhOr345#10:0.12616653468319743991,LhOr346#9:0.15424069
095171796806)#11:0.07241158011235249159)#12:0.03025773270422742536)#13:0.0634585236414935
4163,((LhOr347#14:0.10093178336060988631,PbOr272#15:0.13033369830517391175)#16:0.06391302
149997249438,(PbOr279#17:0.16173766888248350404,((LhOr338#18:0.12623919498492863767,PbOr2
70#19:0.17425320213594025498)#20:0.05229234001437480656,((LhOr341#24:0.01579422594281186 8
78,(LhOr340FIX#26:0.01394266075224939901,LhOr339FIX#25:0.02378371394374283881)#27:0.00243
589059045940665)#28:0.13110017576376253867,(LhOr342#21:0.05065382363233122270,LhOr343#22:
0.06654941384567342189)#23:0.18842948248339330686)#29:0.02665653959142357671)#30:0.029343
18269573192584)#31:0.09467835410266642326)#32:0.04612386456252177275)#33:0.08167595351471
539089)#34:0.20961376214226837278)#36:0.27210174528807190564)#39;
```

Clade 10:

```
(LhOr233#104:0.10026888552919549502,PbOr383#0:0.11943124711136463012,((PbOr231#98:0.07195
20137713067636 8,(PbOr232FIX#100:0.07633772493638474321,LhOr350#99:0.10059368319248487844)
```

#101:0.03744469582922363426)#102:0.11560088353420214236,(LhOr325#96:0.2411493179765144545
5,((LhOr316#90:0.15455975288313530958,(PbOr319#91:0.08583022796349082384,LhOr315#92:0.143
01453206068850954)#93:0.08700966678505345220)#94:0.04534329891829318171,((PbOr294#84:0.20
840268913527429384,(LhOr324#86:0.07721886350527594478,PbOr299#85:0.07979688766777880138)#
87:0.14971258086454808378)#88:0.04351323041425214644,((LhOr333#1:0.30932238016513030621,(
(LhOr354#20:0.13840933433842239619,PbOr287FIX#19:0.18930929514576572137)#21:0.0784095655
0818703463,(LhOr319#23:0.12032318270849427788,LhOr318#22:0.13318624840150242172)#24:0.139
31353284280548199)#25:0.05125290593315037085,((((PbOr292FIX#14:0.00833634056870910696,PbO
r293FIX#13:0.01415437066231744687)#15:0.09450926653711590186,LhOr320#12:0.111335316485241
93358)#16:0.13398521862598483656,((LhOr322#7:0.09522595633032165940,PbOr295#8:0.095731728
10042582248)#9:0.10183749861051004382,PbOr283FIX#10:0.22990740482586377591)#11:0.07854849
154217806440)#17:0.02398003519893597210,((PbOr297#3:0.09434732590336308844,LhOr323#4:0.12
037979718851710143)#5:0.13408953191526715165,PbOr296#2:0.28256285312436668633)#6:0.052322
22848568847395)#18:0.01982654685491656854)#26:0.03034697747126048617)#27:0.02397350037206
704559,(((((LhOr334#64:0.07986733452574333592,PbOr309#65:0.09566414377714232975)#66:0.031
80255187739687572,(PbOr315#67:0.03774372158057306970,((PbOr312FIX#69:0.003271217178000601
11,PbOr313FIX#68:0.00356005264607229524)#70:0.02335631606073828789,(PbOr314#72:0.01157164
769912470519,PbOr311#71:0.02379473000342737996)#73:0.00820647603499710665)#74:0.065282771
70162924414)#75:0.06033369441751949142)#76:0.10491433095410691856,(PbOr316#77:0.103370262
71817575940,LhOr336#78:0.14659334060310028058)#79:0.11804099519966340659)#80:0.0418558263
2964192362,(PbOr320#59:0.16058255207004570875,(LhOr314#60:0.17440164735673382990,LhOr313#
61:0.18076096815027364872)#62:0.05258239714140428706)#63:0.08598345134378024279)#81:0.025
90640025241095629,(LhOr317#28:0.18438243916921109866,(((PbOr302FIX#30:0.01322281490356184
627,PbOr301#29:0.01534543350682927507)#31:0.05786170862975911766,LhOr326#32:0.08420301318
835636539)#33:0.12303305602833532195,(PbOr303#34:0.18583779169833905542,((LhOr332#35:0.09
460963499216952510,(PbOr307#36:0.04939755928605480545,PbOr323FIX#37:0.0512307948346957392
5)#38:0.06413196714124407005)#39:0.03842482281597585370,((PbOr304FIX#49:0.059204382990811
76581,(PbOr322#50:0.03388814992264089360,PbOr305#51:0.04948388639518658261)#52:0.02359562
879777822586)#53:0.04787201333583579466,(LhOr331#40:0.08723588540366809807,(LhOr327#46:0.
04818572212264995991,(LhOr328#41:0.04564622210461836277,(LhOr330#42:0.0422236297978672084
4,LhOr329#43:0.04543517756279642772)#44:0.02322449987296213575)#45:0.02495128683689484470
)#47:0.02154278971979128876)#48:0.02223426128770717922)#54:0.04404208256287357737)#55:0.1
2671527823050776629)#56:0.04591138382871283452)#57:0.02891381969374324026)#58:0.021566032
73030296281)#82:0.03548475754700645274)#83:0.03484020336698994552)#89:0.02115134727741501
547)#95:0.04786207166171323885)#97:0.22284903716036391641)#103:0.10062646770817308683)#10
5;

Clade 11:

((LhOr268#0:0.02863297821869928428,LhOr269#1:0.03807838287175410730)#2:0.1204860593618709
7021,LhOr264#148:0.16932600071202255632,((LhOr273#3:0.13361363019735783553,((LhOr280#6:0.
03583575761435609658,LhOr272#5:0.07419757186859761433)#7:0.07405189645970484957,LhOr271#4
:0.19657554624452636127)#8:0.02699228413793333323)#9:0.03926461058816243382,(((LhOr266FIX
#10:0.05013227435699574297,LhOr267#11:0.06966143614055107791)#12:0.11415822886070307873,(
LhOr274FIX#13:0.10808478220261906466,((LhOr275#20:0.03171055070381569724,LhOr276FIX#19:0.
03490800146541288979)#21:0.10177273753552402991,(LhOr277#14:0.09013900802943551616,(LhOr2
78#15:0.06208380451233402181,LhOr279#16:0.06490502677472920201)#17:0.09753037794624232204
)#18:0.04970068698979328986)#22:0.02889564241037491377)#23:0.11989010345447483064)#24:0.0
4279756616960202903,(((PbOr395FIX#29:0.16730872384112091544,(PbOr393#26:0.114078230908815
71981,PbOr394#27:0.12102367281326585624)#28:0.08024149459544548113)#30:0.0231632276627846
2036,PbOr391#25:0.23539277731323382525)#31:0.03445085802002299802,(((((LhOr292#40:0.13538
445674815094422,(LhOr291#41:0.03119440417307267621,(LhOr290FIX#42:0.02405482222317750773,
LhOr289FIX#43:0.02752865587475598549)#44:0.02390681227128260700)#45:0.1305936796776337172
1)#46:0.05367713325445729738,(LhOr288FIX#37:0.18446162180163822031,LhOr293#38:0.196147501
00857314408)#39:0.04975882803002023430)#47:0.01936090144419236325,(LhOr284#32:0.209422059
04189911680,(LhOr281#33:0.09573618858845478474,LhOr282#34:0.15881820812447913771)#35:0.07
707444279580065449)#36:0.03017588994268752614)#48:0.04101100123519996199,((lhOr286#50:0.0
4965435077709723366,LhOr285#49:0.08767985197384688123)#51:0.17237261787685506653,((PbOr32
7#59:0.13548695658280729415,(PbOr325FIX#61:0.10666743554421743989,PbOr326#60:0.1687030356
3853902840)#62:0.07449531232052020169)#63:0.05624238933943424140,(PbOr333#52:0.1430311276
8455769621,((PbOr331#54:0.08572296284604143113,PbOr329#55:0.10661762641260459639)#56:0.02
758750759043292547,PbOr330FIX#53:0.15309611687641538902)#57:0.09479455466282606102)#58:0.
08844632860419450460)#64:0.03357548271098467763)#65:0.02512547816708526893)#66:0.06495655
541030564972,(((((PbOr364#114:0.01658659332148169560,PbOr363FIX#113:0.0309760669323305634
6)#115:0.04973051362120155250,(PbOr362#110:0.01724928979380072058,PbOr358#111:0.032736330
07685351065)#112:0.04839231336467382516)#116:0.05496821670661840992,(((PbOr353#122:0.0175
5561445272167675,PbOr355#121:0.01877540102370938921)#123:0.02466703356076831513,PbOr357FI
X#120:0.05287947595110589760)#124:0.05451100586671155795,(PbOr350FIX#117:0.00760763869244
039052,PbOr351FIX#118:0.01051852814136977621)#119:0.10473719409150214488)#125:0.079993851
57698424287)#126:0.11838455548395866723,(((LhOr310#134:0.12504429397033439697,LhOr311#135
:0.13055956301777038586)#136:0.04559889912223837050,(PbOr365FIX#138:0.1512622099207519554

8,PbOr367FIX#137:0.18034726803397235773)#139:0.03534595260178934051)#140:0.03202142179843
234931,(LhOr312#127:0.25630906241464956885,(PbOr399#128:0.19274152310197043003,(PbOr398FI
X#130:0.07779248543002517668,PbOr396FIX#129:0.08432771885045979576)#131:0.14445369865151841404)#132:0.07354404719742239915)#133:0.03609529006921920191)#141:0.02863641799748165712
)#142:0.03916312563275579223,((((PbOr339#77:0.11847818038584242983,PbOr338#76:0.145119702
62673351550)#78:0.09283742223739958888,(LhOr304#79:0.08409850868505480692,(LhOr302#80:0.0
313038869345887357 8,(LhOr303FIX#81:0.05526108674466199272,LhOr301#82:0.06820526978456525891)#83:0.01332619600439463765)#84:0.10572793289415266527)#85:0.11616626173833383140)#86:0
.03666905085243328533,(((LhOr307#98:0.02841502658148944288,(LhOr306#99:0.0229967255363151
2337,LhOr305#100:0.02780873893601139638)#101:0.01345138432873636825)#102:0.10201054917775
405351,(LhOr309#104:0.04266656664312228758,LhOr308#103:0.04678908201318494192)#105:0.1293
935465173639554 1)#106:0.08237634205785901442,(PbOr340FIX#87:0.19249885031452709794,(PbOr3
41FIX#95:0.09056935556997137982,((PbOr347#91:0.08337634693332766911,PbOr346#92:0.09603743
82525422953 8)#93:0.03434354807601758958,(PbOr342#89:0.09893631615791781408,PbOr344#88:0.1
17791695992445266 67)#90:0.03431678117838254938)#94:0.04370686905461610566)#96:0.079515033
04466016331)#97:0.03625587772185622643)#107:0.07476797065062266989)#108:0.027590132571346
32427,(PbOr337#67:0.21492604917155991306,(LhOr300#68:0.12316930196422916366,(LhOr297#72:0
.13858498339503541597,(LhOr298#69:0.07124126014892012693,LhOr296#70:0.104334498884298673 6
9)#71:0.06120211332537308507)#73:0.03349446055839892106)#74:0.11808226352264125647)#75:0.
11061964464731882662)#109:0.02779909024404219817)#143:0.02525186703369305605)#144:0.07299
48298503684117 6)#145:0.03338558849033156778)#146:0.01590629294164431010)#147:0.0329256371
8897435071)#149;

Clade 12:

(NvOr294#0:0.14159872467648079719,NvOr293#76:0.16453526157575129596,(NvOr295#74:0.1887954
9976327444916,(NvOr61#72:0.24958763999852243143,(NvOr41#1:0.30217893556259289856,((NvOr60
#68:0.23652733065499143006,LhOr158#67:0.27441564875636526022)#69:0.07414021469656380259,(
((LhOr146#3:0.07511238548539500570,PbOr52#2:0.08523243584458076172)#4:0.22635597006331348
302,((LhOr147#6:0.05686967463858374344,PbOr53#5:0.07133862663659087944)#7:0.1192856941437
6273550,(PbOr54FIX#8:0.09727974230879245232,(LhOr150#10:0.04820262820932088438,PbOr56FIX#
9:0.08626163142386328309)#11:0.05332367855484879127)#12:0.10835199539931381074)#13:0.1076
945057273515360 4)#14:0.06632321187153077113,((((LhOr165#33:0.05392055515893178635,PbOr67#
32:0.08305337624708959698)#34:0.06461430108938488737,((LhOr159#41:0.07359338363496840951,
PbOr64#40:0.07795320054019029554)#42:0.02529105741648814826,(LhOr157#35:0.106080013407863
35498,(PbOr63#37:0.07850437469905570398,PbOr58#36:0.09021229354658318045)#38:0.0224704911
4323653590)#39:0.02223893393336068627)#43:0.08541845725821252400)#44:0.069596810302175862
73,((PbOr68#29:0.04963198066419766824,PbOr69#28:0.05935082214439147547)#30:0.087810058482
17886267,(LhOr160#15:0.08717791579722818096,(LhOr170#16:0.09241660522677636047,((LhOr163#
20:0.09822963458150404947,(LhOr162#22:0.05985464842908847644,LhOr161#21:0.086502220492316
70596)#23:0.01721394015257243781)#24:0.01337872665498743806,(LhOr168#17:0.042055769075085
04214,LhOr167#18:0.04467310923476502244)#19:0.08150731410622685802)#25:0.041791543445216 3
1564)#26:0.02561697367192869446)#27:0.04663122056440573843)#31:0.06146872476739072277)#45
:0.15273729213582673547,(LhOr156#63:0.23465795191941685172,((PbOr66FIX#55:0.1324229880545
2040744,(LhOr151#53:0.05109357349650206492,(LhOr152#46:0.02202180928329261489,(LhOr153#47
:0.03058981877151869741,(LhOr154#49:0.01682417341099870511,LhOr155#48:0.02186217313213462
479)#50:0.01499641932871122879)#51:0.00745374082210316109)#52:0.03720477754946218546)#54:
0.08793644688847378210)#56:0.10706195495827677822,(PbOr65#60:0.08887628642564660009,(PbOr
55#57:0.08574017074322998200,LhOr149#58:0.09575566198486880343)#59:0.04270620197362323678
)#61:0.21621051630460616511)#62:0.10403253700889469879)#64:0.05018006432528555882)#65:0.0
4659359999061952334)#66:0.09887478443638904324)#70:0.07683011620272019171)#71:0.098342113
56539013238)#73:0.38744395307800272299)#75:0.06825886179606041604)#77;

Clade 13:

(NvOr62#170:0.11308516969430117138,(NvOr71#167:0.06257026274467908378,NvOr72#168:0.063890
69158569699036)#169:0.12684795614847121925,(NvOr64#0:0.18129251566872758850,(NvOr65#1:0.1
4250997447183347266,(((NvOr68#3:0.13387574411152633469,NvOr69#2:0.13603483110998160210)#4
:0.04382626797347222158,(NvOr67#6:0.12646962023937188824,NvOr66#5:0.14265200463269434028)
#7:0.04352541838872611701)#8:0.12891584716701351421,((LhOr71#9:0.07908718294446694108,PbO
r104#10:0.09686505834210758226)#11:0.12001627737667265594,((LhOr74#12:0.05300928804888768
026,PbOr107#13:0.08959915965438558505)#14:0.14489654727400111400,((LhOr96#16:0.0986818412
3087871129,PbOr122FIX#15:0.11151296629110694913)#17:0.11361278242293504359,((((LhOr73#95:
0.05809130994440350748,PbOr106#96:0.08838747200022109562)#97:0.13132925261560285390,(LhOr
72#99:0.07412007475434589931,PbOr105FIX#98:0.08967631849019770673)#100:0.163985048875466 6
9351)#101:0.05966236963806022947,(((LhOr75#133:0.05542825555520979747,PbOr108#134:0.07162
42556029181021 1)#135:0.13046312156454492603,((PbOr120#136:0.17882364961338445553,(PbOr121
#137:0.08586022449009408919,(LhOr95#138:0.02682138153199893973,LhOr94#139:0.0326541100890
0377665)#140:0.07986039741072932518)#141:0.08710389355395155297)#142:0.06396575084458629 1
57,(((PbOr109#152:0.07062050179915652093,LhOr76#151:0.08508418047433674580)#153:0.0283783

6879822810271,(PbOr110#148:0.08474143422998055286,LhOr77#149:0.10053277115884261261)#150:
0.05498623352625489624)#154:0.03198605302960472879,(LhOr78#143:0.09033713286921089824,(Pb
Or111#145:0.00487808264248340909,PbOr112#144:0.01710848603673818802)#146:0.10695197218195
406919)#147:0.07273266024578448063)#155:0.08365181397735474422)#156:0.0273771920271268752
5)#157:0.02372717990096075494,(((PbOr114#129:0.07419560971195973686,LhOr80#128:0.07449269
271214631338)#130:0.03921903919144634904,LhOr79#127:0.12718465001532625047)#131:0.0698850
2625811028601,(LhOr85#102:0.09472014064840440783,(LhOr81#124:0.08541739873791071025,((((L
hOr86#103:0.10347099915547282312,LhOr92#104:0.10921502868895371918)#105:0.019723618099925
78818,LhOr89#106:0.14316241805139770515)#107:0.01351337718328749458,((LhOr87#114:0.092675
53623575182364,LhOr91#113:0.09385899146089424216)#115:0.01940780280793059920,(LhOr84#108:
0.09183678530815671259,(LhOr83#109:0.08652512987390398314,LhOr82#110:0.088753487685501328
27)#111:0.03019407121859854146)#112:0.05617172726688340889)#116:0.01429405967660748143)#1
17:0.01380211947107188447,((PbOr115#120:0.07457736270803659584,PbOr116#119:0.082451066052
42023823)#121:0.02516996322333782052,PbOr118#118:0.14977511710628124697)#122:0.0576767562
4528537798)#123:0.01105476436127883315)#125:0.02652587890207258603)#126:0.163469906362223
38833)#132:0.07034172089845409925)#158:0.05959968610369913156)#159:0.02560690686172983205
,(((LhOr116#26:0.06186219443304359633,PbOr143#25:0.08439999402940603912)#27:0.11455572783
041090279,(LhOr97#18:0.09407746617168268044,(PbOr125#22:0.11317593706346353033,(PbOr123FI
X#19:0.00052792749707240437,PbOr124FIX#20:0.00211577659493229417)#21:0.113020487610510977
92)#23:0.04236399354735360251)#24:0.15341586829756298505)#28:0.03431510710658299684,((PbO
r130#29:0.09349840906727022172,LhOr102#30:0.11266564413750410067)#31:0.128892118313187475
92,(((LhOr100#39:0.07410285716280938517,PbOr128#40:0.09726396335378191538)#41:0.059073007
51830724117,((LhOr99#36:0.06746218278037467708,LhOr98#35:0.07948889139348086497)#37:0.021
26018486421387549,(PbOr127#33:0.06834877672387609793,PbOr126FIX#32:0.07542850278625155835
)#34:0.05197608571258643256)#38:0.09191449832867318315)#42:0.09202966894303570011,(((LhOr
104#83:0.11920792802578028535,PbOr131#82:0.12817766862571924880)#84:0.0639429866380496758
6,(LhOr103#88:0.17524911328406081457,(PbOr129JF#86:0.08769582149970496510,LhOr101#85:0.11
882451800817882270)#87:0.06181342917333672304)#89:0.04250059068240088361)#90:0.0533723705
0467650429,(LhOr105FIX#43:0.13120048427178293560,((((LhOr114#73:0.00727591022794480281,Lh
Or115#72:0.00921091314941304036)#74:0.00791973296028001747,LhOr113#71:0.02033326077945010
399)#75:0.10034652721435059763,(PbOr144#76:0.06801466169813279172,PbOr142FIX#77:0.0815627
8282146152414)#78:0.09977274572823525978)#79:0.07086573889273127536,((LhOr108#67:0.071650
02950916457558,PbOr133#68:0.08725688935490120979)#69:0.07298961994870245551,((LhOr112#64:
0.07032239942354323592,PbOr141#63:0.10998269966790810703)#65:0.05161353221498058558,((LhO
r111#47:0.10703525084627772690,(PbOr140#45:0.04169694109027122925,PbOr139#44:0.0445585255
4900206069)#46:0.08181618273882200476)#48:0.07445329908624365167,(PbOr138#49:0.1601076206
8528244795,((LhOr109#57:0.06016172639968349334,LhOr110#58:0.10463741549305008438)#59:0.02
523143543401113603,(PbOr134#50:0.04440151252159974665,(PbOr135#51:0.04573561948093250035,
(PbOr136FIX#52:0.01563483488267221680,PbOr137#53:0.02335259136648434181)#54:0.06692674475
298181214)#55:0.02998599504936700147)#56:0.05532047456699956678)#60:0.0208000459514716372
3)#61:0.03194094140267877968)#62:0.02075945193170779940)#66:0.01630126438938846178)#70:0.
01663752238739617945)#80:0.04030284977806333074)#81:0.11661070090273742417)#91:0.05196845
207350933582)#92:0.03079014626850738734)#93:0.04917085148764889502)#94:0.0446252221988241
6458)#160:0.02848824429860844290)#161:0.03795197235818736914)#162:0.08032505707183343335)
#163:0.30650416408991809636)#164:0.21003987801002751978)#165:0.05710601967966152243)#166:
0.07746163119346025083)#171;

Clade 14:

(NvOr298#22:0.19005564634947333391,NvOr299#21:0.33621983118868387086,(LhOr205#0:0.3481380
5766426531019,((PbOr146#16:0.13011079286996729554,LhOr206NTE#17:0.14620189395838065205)#1
8:0.19912986285970668554,((LhOr207#1:0.06763695391146691549,PbOr148#2:0.09084226673010761
810)#3:0.25108356648207391659,((NvOr289#12:0.20350348734688025742,(LhOr208#10:0.067837706
99301336360,PbOr149#9:0.09426446173347946544)#11:0.13416407690090606519)#13:0.09196531960
174476883,(NvOr288#4:0.28429906753861922120,(NvOr292#6:0.04827160608298006195,NvOr291#5:0
.09629611527230363954)#7:0.26745154641425822328)#8:0.09528218550122788399)#14:0.080205464
09463776005)#15:0.07068917811591245159)#19:0.06382492637248762812)#20:0.26358828066340045
382)#23;

Clade 15:

(PbOr77NTE#0:0.05792297001662759237,LhOr172NTE#142:0.08831890085221165421,(PbOr76NTE#140:
0.08436100988109158927,(((LhOr203NTE#136:0.07920623091191622556,(PbOr102NTE#134:0.0253372
0716879305640,PbOr101NTE#133:0.02877708848023225255)#135:0.07024546795574812541)#137:0.12
010280309406526356,(((LhOr173NTE#125:0.06202675725317112476,PbOr92NTE#126:0.0632409158835
1238302)#127:0.10176323863978498085,(LhOr199FN#128:0.01018854931993059577,LhOr201NTE#129:
0.01192839100089270604)#130:0.15382707811374812801)#131:0.03047634260959491623,((LhOr202N
TE#95:0.18844566573473367499,((LhOr195NTE#101:0.08142016782225212912,(LhOr200NTE#96:0.011
55744880136531927,(LhOr198FN#97:0.00788657870731213073,LhOr196NTE#98:0.011011063604891234
08)#99:0.01444989305812833906)#100:0.11464726208905072491)#102:0.05584584957867873983,(Lh

Or192NTE#103:0.15342175725314391443,((LhOr194NTE#117:0.11216330998241010042,LhOr193NTE#11
8:0.11858685155244778897)#119:0.02214520708711763264,((LhOr186NTE#104:0.0630914744912O475
204,(LhOr191FN#105:0.07888033717745595 36,LhOr187FN#106:0.09339675877494044154)#107:0.022
7524947140120559 2)#108:0.02243849619062475084,(PbOr84NTE#109:0.08120630733951163249,(PbOr
85NTE#113:0.06263616653284509450,(PbOr80FN#110:0.02835579794729253811,PbOr79FN#111:0.0361
152532760504740 8)#112:0.04682744770212756563)#114:0.03774606610645048210)#115:0.020684618
92972643656)#116:0.04808170289192485042)#120:0.01925132639367344023)#121:0.02428357520056
597871)#122:0.07881453910134704477)#123:0.03551252408190237048,(LhOr185NTE#70:0.091397063
90677478087,((LhOr174NTE#72:0.06829172086054735580,PbOr93NTE#71:0.07662869697183456730)#7
3:0.03058035805285200942,((LhOr184NTE#74:0.02129006047789142736,LhOr182NTE#75:0.043018991
79902504006)#76:0.05143766439955559794,(LhOr175NTE#77:0.07743341949279816672,((LhOr183NTE
#79:0.01302354425898463557,LhOr181NTE#78:0.01412470150454097489)#80:0.061404860817920146 8
9,(LhOr178FN#81:0.03884714859482228666,((LhOr179JFN#82:0.01536367019108257279,LhOr180FN#8
3:0.02937284834987576032)#84:0.00592681081934937765,(LhOr177NTE#86:0.01981522384058501812
,LhOr176NTE#85:0.02338223339995399580)#87:0.01672992978119570637)#88:0.00923383562965691 4
41)#89:0.05941621020005746767)#90:0.02262815874446749698)#91:0.05464577280890953875)#92:0
.03714402562833796911)#93:0.02315989874077650085)#94:0.11490282662264766911)#124:0.027168
21428162348451)#132:0.04420259291847369182)#138:0.03629049243171369316,((NvOr38#1:0.20739
24130584803016 1,(NvOr35#2:0.10832925329628927125,(NvOr36#4:0.06707780883058439558,NvOr37#
3:0.09081931816972528548)#5:0.11963934994928725197)#6:0.08156466618730506823)#7:0.1630619
703938678832 8,(((LhOr69NTE#62:0.04380242946878087212,(PbOr74NTE#63:0.01582453184718538020
,PbOr73NTE#64:0.03368735874433777255)#65:0.06569675606659634848)#66:0.19287630274327322 02
2,((LhOr70NTE#58:0.07481559641465927724,PbOr75NTE#59:0.09424529179350511765)#60:0.1718618
271559312904 5,(PbOr71FN#43:0.09999037454687921544,(PbOr72NTE#44:0.10518945295009879404,(L
hOr60NTE#54:0.06949955131787961815,(((LhOr63NTE#49:0.00066584938776774179,LhOr61NTE#48:0.
0047892369359050527 8)#50:0.04659730374775940959,LhOr64NTE#51:0.05217582697562132138)#52:0
.01590733510045756749,(LhOr66#46:0.03150059981692060473,LhOr68NTE#45:0.04425337510975680 5
38)#47:0.05359429243030915807)#53:0.01483901860763635686)#55:0.01978056233601944991)#56:0
.03239700547461106528)#57:0.18122043218066283266)#61:0.06068723902466284514)#67:0.0599202
349082727861 5,(((NvOr28#31:0.13779720453288551996,(NvOr29#33:0.10412671617023804005,NvOr3
1#32:0.17105629829895693805)#34:0.05728191686640998065)#35:0.05480260841115020681,(NvOr24
#36:0.20305478061915027688,(NvOr25#37:0.11153055663249457352,NvOr26#38:0.165396154855478 7
8859)#39:0.08645929485014544613)#40:0.04453638456705404786)#41:0.13431476507017212074,(((
(NvOr20#16:0.12139099475971937281,NvOr19#17:0.13046920734375108686)#18:0.079995362789483 8
6758,NvOr15#15:0.21518329776552777566)#19:0.09710074560235230912,((NvOr14#25:0.184322630 2
7362261187,NvOr13#26:0.25094508544430504937)#27:0.04261331116284578641,(NvOr17#23:0.12272
01536157862121 8,(NvOr12#20:0.06676677696807342688,NvOr16#21:0.07721536288778936552)#22:0.
1515991253323274168 3)#24:0.06824105316534746779)#28:0.04817395590085615797)#29:0.04284908
39886018215 5,((NvOr27#8:0.12242018717984047560,NvOr23#9:0.14559718422681347527)#10:0.1504
642724689929100 8,(NvOr22#12:0.17747042591341760653,NvOr21#11:0.25132512758363595440)#13:0
.05771800262819634653)#14:0.10033134107355362596)#30:0.05458266841251473850)#42:0.0604753
323632114328 5)#68:0.07008321273266669704)#69:0.14014988224512736625)#139:0.07745700581612
842417)#141:0.06898845141172013640)#143;

Clade 16:

(PbOr19#177:0.08508821311292545653,LhOr21#178:0.10063793004694067401,(((PbOr24#0:0.044812
8081910293893 4,PbOr25#1:0.04510290668744612597)#2:0.12855287846272672736,((LhOr23#3:0.008
1371239168935728 0,LhOr25#4:0.02617908056292785057)#5:0.06757516088380703667,(PbOr20#6:0.0
774828332687980680 9,PbOr23#7:0.14527149778917111678)#8:0.02953499989983601748)#9:0.035449
1129286874165 3)#10:0.09321653530086784767,((((LhOr39#16:0.07657415574521615753,PbOr37#17:
0.10484838326185097479)#18:0.10417583963013607451,(LhOr40FIX#11:0.09948344930305391687,(P
bOr40#12:0.09148817912418338860,PbOr38#13:0.11569181255943312980)#14:0.049995727360838189
41)#15:0.08531661711502125378)#19:0.08810747008850033923,(((LhOr26#56:0.08201269057550951
425,PbOr21#57:0.09215383056055889699)#58:0.12212935793440195253,((PbOr33#44:0.17154312574
357366561,(LhOr30#42:0.07637282295844541324,PbOr28#41:0.08903695198267193300)#43:0.089844
49621834446342)#45:0.03987241359933949014,((PbOr27#46:0.07435022368403622350,LhOr29#47:0.
09349161851610551799)#48:0.11727030772057756014,(PbOr26#49:0.08817037784434593461,(PbOr22
#51:0.06512356345620586018,LhOr27#50:0.09184549735982269758)#52:0.03060903293736276856)#5
3:0.10977216223273164597)#54:0.03351513905986234360)#55:0.02301007068682352219)#59:0.0265
564812910581322 9,(((PbOr32#37:0.06622185832675753225,LhOr34#36:0.07424687176349335782)#38
:0.02571159148265235450,(PbOr31FIX#34:0.05058108661904277270,PbOr30#33:0.059618355516040 5
1375)#35:0.06800306102379093109)#39:0.03720166405456085817,((LhOr37#21:0.080783265504465 6
2893,PbOr36FIX#20:0.12079896285405630763)#22:0.05647754509199947454,(LhOr38#23:0.13367630
60992932905 5,(PbOr34#24:0.09341616859804252604,(LhOr35#28:0.04259684184365548909,(LhOr32#
26:0.05195927381773565029,LhOr33#25:0.05906474025792859267)#27:0.14425526218644663112)#29
:0.04505704416038316257)#30:0.04310138895706434187)#31:0.03733830205488337939)#32:0.04704
7295490688763 32)#40:0.10209397317331087018)#60:0.04517549334518947829)#61:0.0823576559296
6295956,((((((PbOr43#127:0.02513733332597735404,PbOr42#126:0.04597657872554086061)#128:0.
0885580689911274615 6,(PbOr44#129:0.08253972340198312274,LhOr42#130:0.08732389083209488034
)#131:0.05559707400862894111)#132:0.09137932689044288292,(LhOr43#133:0.12830609790078464 9

16,PbOr46#134:0.13988913535371566566)#135:0.17352211289944577399)#136:0.05195228151944623
896,((LhOr45#113:0.07655178410787882859,(PbOr48#115:0.08202022362306579661,PbOr47#114:0.0
917635409806832197 4)#116:0.03177288929239235199)#117:0.04410158276656572784,(LhOr46#123:0
.06676908653034788199,(PbOr49#118:0.03344750404966601726,(PbOr50#119:0.04814768905578629 9
65,PbOr51#120:0.05679572002408159875)#121:0.02013075301400072889)#122:0.06937336371672113
422)#124:0.04905241991583712824)#125:0.20339850181996121936)#137:0.06506606342715653968,(
((NvOr5#139:0.28142229248756600590,(LhOr41#140:0.06087896018391263792,(PbOr39#141:0.05720
210240613457509,PbOr41#142:0.06528012647148941083)#143:0.04035444330254587403)#144:0.1942
192028614926346 8)#145:0.06721372180865968493,NvOr3#138:0.42809469895142482354)#146:0.0600
942830205664843 5,((PbOr4#150:0.19077026179876477308,(PbOr5#147:0.09783206334010499661,LhO
r4#148:0.10739142251667260775)#149:0.11093814064127889563)#151:0.21323563928487523911,((N
vOr2#168:0.16787110639274116508,((PbOr2FIX#164:0.05443396364234111273,LhOr2#165:0.0633731
4153777132142)#166:0.04017598829601903149,(LhOr3#162:0.07232988277713264802,PbOr3#161:0.0
8602351370771615935)#163:0.06814392222737826466)#167:0.08753927382062862139)#169:0.203057
74174619203865,((NvOr9#155:0.12371413697905783713,(NvOr6#157:0.11737171799965644137,NvOr7
#156:0.13284277567039823320)#158:0.08921062594623745123)#159:0.06942658138578709781,(NvOr
10#153:0.14492872262006373241,NvOr8#152:0.29254275794174022796)#154:0.06903749355786505 11
5)#160:0.11096708038035126009)#170:0.05460790252378437681)#171:0.04993216767483663748)#17
2:0.04500294221236822434)#173:0.02030095158002284683,(((PbOr18#101:0.07574720500667271594
,LhOr20#102:0.10511811238254548106)#103:0.19520067423868453504,((PbOr17#108:0.01895432632
271175713,PbOr16#107:0.04234428644201301134)#109:0.06811362611180034332,(LhOr18#105:0.025
6849978693469947 8,LhOr19#104:0.06195732050815315473)#106:0.06592397291281737459)#110:0.20
977853097591570020)#111:0.04852146996529031753,(LhOr17#62:0.24082056080189864766,((((LhOr
7#64:0.06662165203526676516,LhOr9#63:0.07513777636342115762)#65:0.04778352298798591719,(L
hOr8#66:0.08000074194485634616,((PbOr10#68:0.03072644635476671354,PbOr12#67:0.03119077980
448724946)#69:0.04752137142825769989,PbOr8#70:0.08228274056465445518)#71:0.03849859517475
068271)#72:0.02219404335170786779)#73:0.05027204377061512269,(LhOr15#74:0.10268276761052 8
09454,(LhOr14FIX#75:0.07061141793481469364,LhOr13#76:0.11573813641680856523)#77:0.0682437
2696336825084)#78:0.07861903298466643708)#79:0.11899465963063966811,(((PbOr6#92:0.0843622
6407436024644,LhOr5#91:0.10784044864167434408)#93:0.10801798029439961535,(PbOr13#94:0.071
58154943854977414,LhOr12#95:0.10251194464489998825)#96:0.14006092415763940484)#97:0.06070
836373421854837,((LhOr16#81:0.08698791141147087236,PbOr14#80:0.09179266124837502649)#82:0
.08335904310634681402,(LhOr6#88:0.14632383585327285447,(PbOr9#86:0.12741008332801964653,(
LhOr10#83:0.05207559580666604182,LhOr11#84:0.06845796861281486234)#85:0.08592211832516460
923)#87:0.09902873777138196387)#89:0.03836447326544246972)#90:0.09935846517202116812)#98:
0.04494903189312279140)#99:0.10599604270141928242)#100:0.04841211025913769173)#112:0.0654
9422589417622231)#174:0.04300868066299180781)#175:0.09192643181901923033)#176:0.136847857
78034441739)#179;

Figure S1

See separate file Sup_Figure_1.pdf

Positive selection in the olfactory receptors gene family. This tree is similar to Figure 3, with Sequence IDs and bootstrap values displayed.

Figure S2

Density plots of expression levels in *Solenopsis invicta* of 313 genes with mitochondrial activity in queens (blue), males (red) and workers (green), at the pupal stage (top) and the adult stage (bottom). Expression was obtained from previously published microarray data (Ometto et al. 2011). Paired *t*-test indicated that the expression of mitochondrial genes was higher in workers compared to queens at the larval stage (p=2.6e-05), and not significantly different at the adult stage (p=0.11). The expression of mitochondrial genes was the lowest in males at both developmental stages (all p-values were below 10^{-6}).

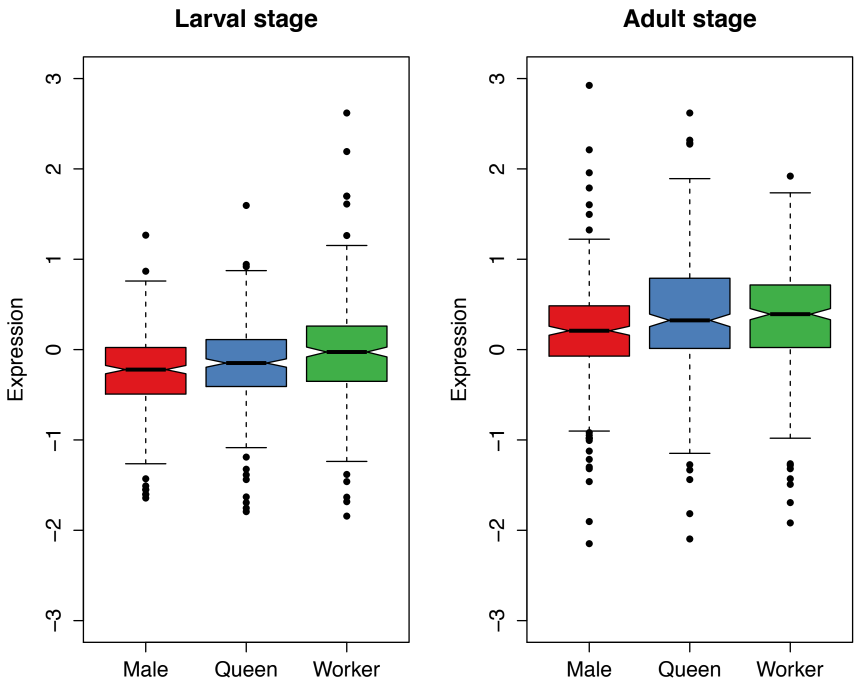

Figure S3

Phylogeny of the five ant species and the four outgroup species used in the ant mitochondrial dataset. Branch lengths are arbitrary. The 11 different branches where positive selection was tested are labeled and highlighted in red. Legend as in Figure 1.

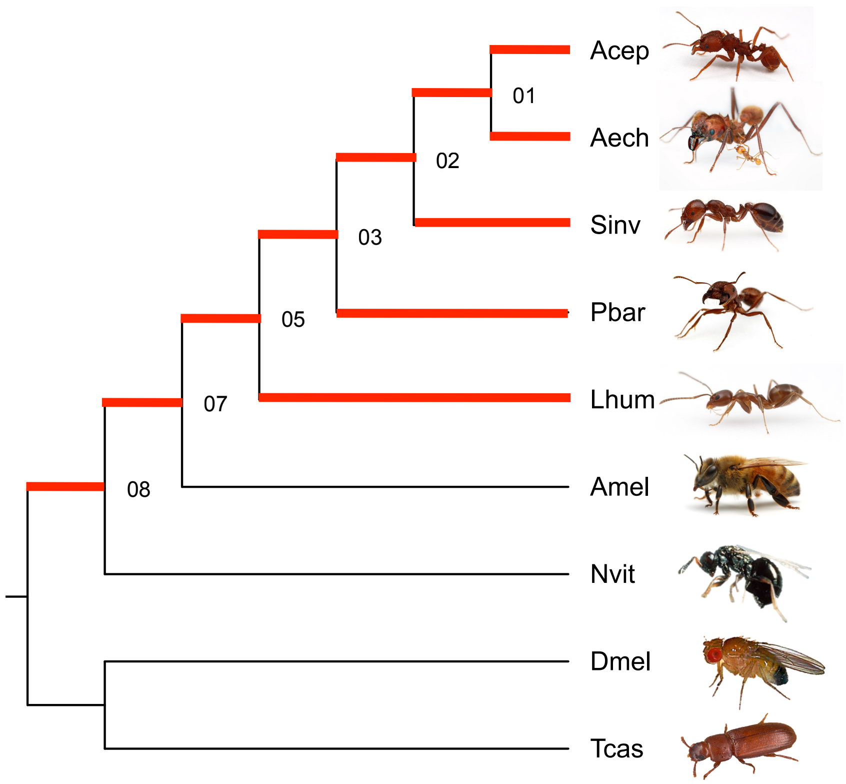

Figure S4

Phylogeny of the twelve Drosophila species dataset. Branch lengths are arbitrary. All branches were tested for positive selection and branch labels are shown. Legend as in Figure 1.

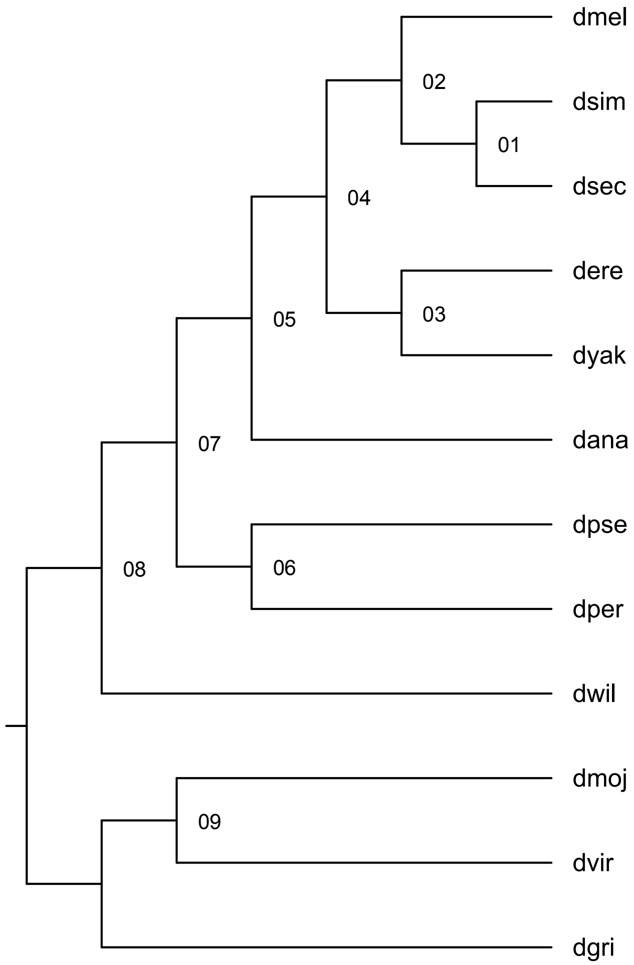

Figure S5

Phylogeny of the ten bee species dataset. Branch lengths are arbitrary. All branches were tested for positive selection and branch labels are shown. Legend as in Figure 1.

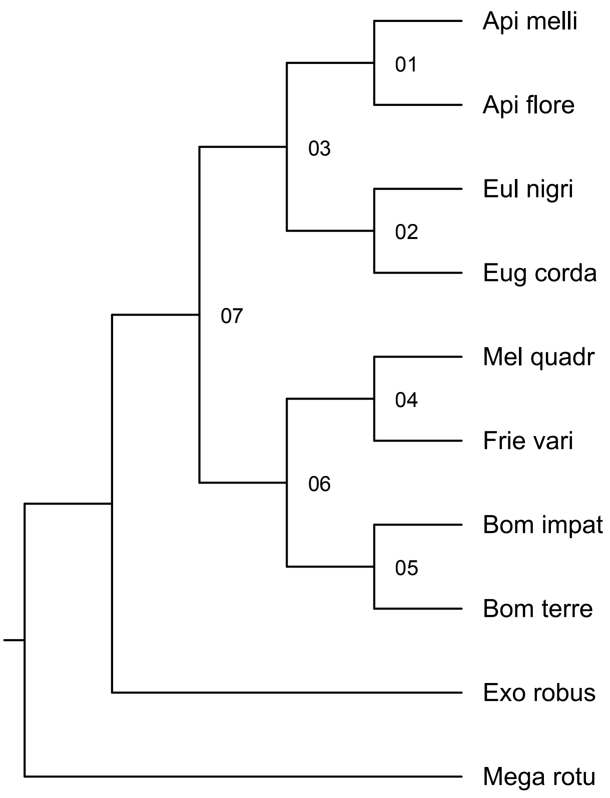

Figure S6

Density plots of SUMSTAT scores of random gene sets calculated on 10,000 randomized gene sets of size n=11 (minimum set size used in our study; left), n=25 (middle) and n=100 (right) are shown in black. The distribution of these scores closely approximates a normal distribution (shown in red) of mean equals n times the mean score of all genes and variance equals n times the variance of scores of all genes.

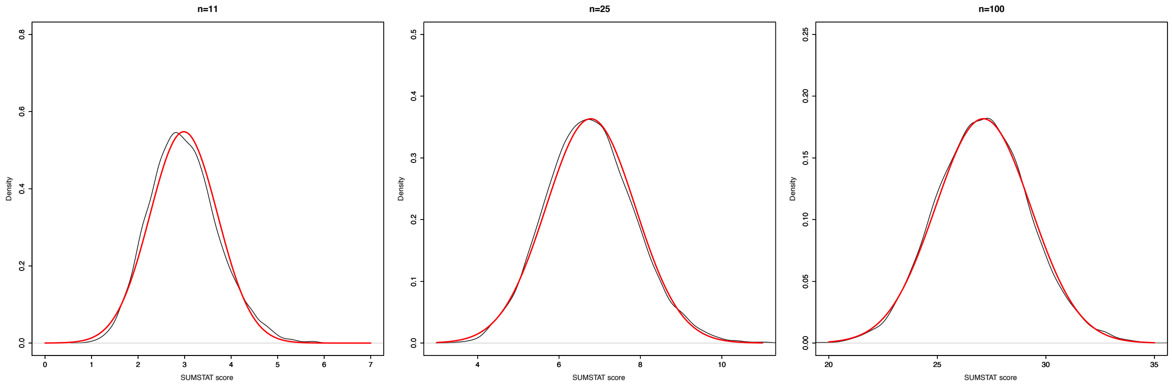

Figure S7

Distribution of scores in Gene Ontology functional categories enriched for positively selected genes. Density plots of scores of genes belonging to individual GO categories are plotted in red and are compared to density plots of scores of all genes analyzed in blue.

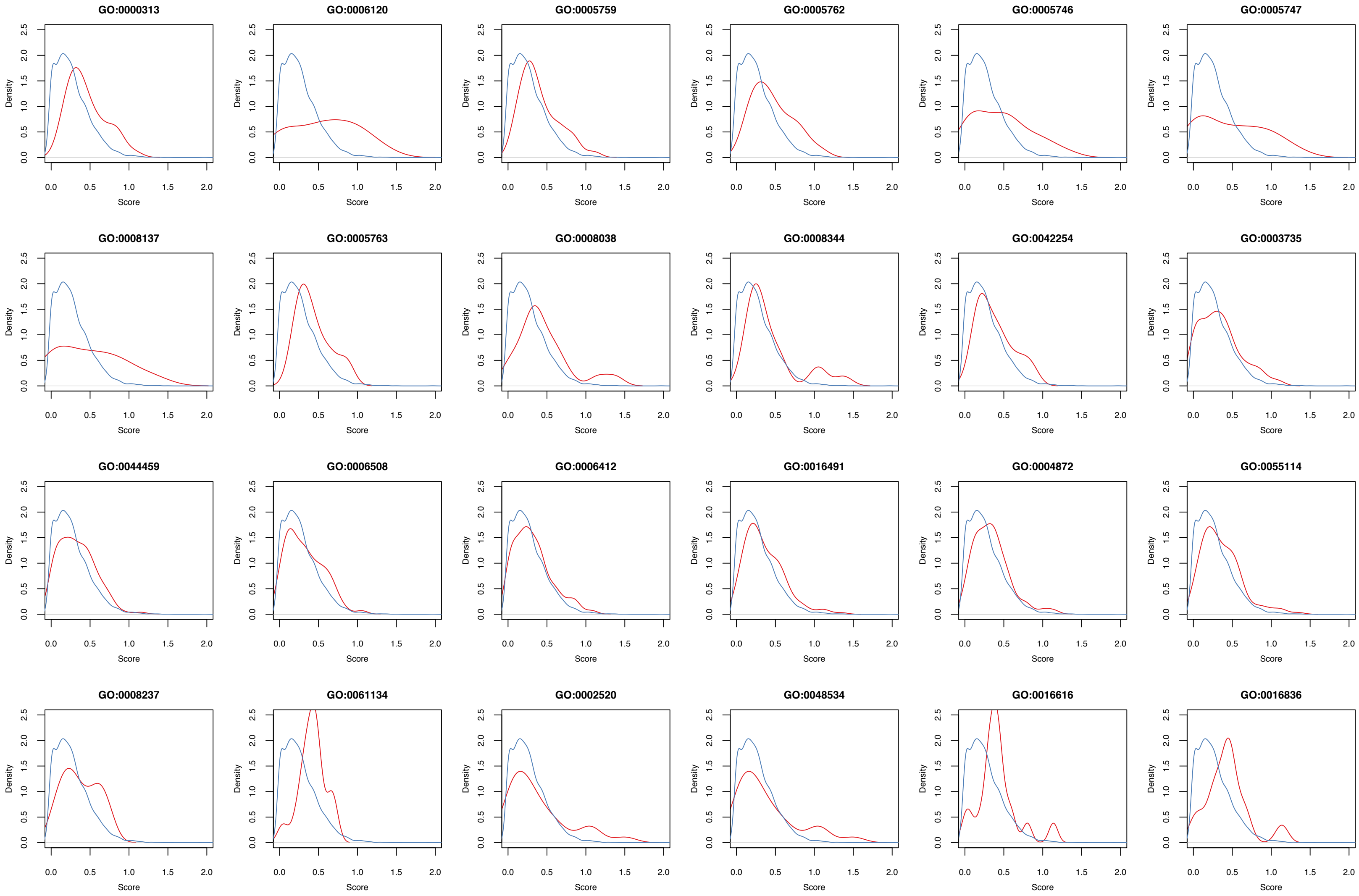
GO:0000313
GO:0006120
GO:0005759
GO:0005762
GO:0005746
GO:0005747
GO:0008137
GO:0005763
GO:0008038
GO:0008344
GO:0042254
GO:0003735
GO:0044459
GO:0006508
GO:0006412
GO:0016491
GO:0004872
GO:0055114
GO:0008237
GO:0061134
GO:0002520
GO:0048534
GO:0016616
GO:0016836
Density
Score
0.0 0.5 1.0 1.5 2.0
0.0 0.5 1.0 1.5 2.0 2.5

References

Drosophila 12 Genomes Consortium. 2007. Evolution of genes and genomes on the *Drosophila* phylogeny. Nature 450:203-218.

Duret L, Galtier N. 2009. Biased Gene Conversion and the Evolution of Mammalian Genomic Landscapes. Annu Rev Genomics Hum Genet 10:285-311.

Fletcher W, Yang Z. 2010. The Effect of Insertions, Deletions, and Alignment Errors on the Branch-Site Test of Positive Selection. Mol Biol Evol 27:2257-2267.

Galtier N, Duret L, Glémin S, Ranwez V. 2009. GC-biased gene conversion promotes the fixation of deleterious amino acid changes in primates. Trends Genet 25:1-5.

Gharib WH, Robinson-Rechavi M. 2013. The branch-site test of positive selection is surprisingly robust but lacks power under synonymous substitution saturation and variation in GC. Mol Biol Evol 30:1675-1686.

Jordan G, Goldman N. 2012. The effects of alignment error and alignment filtering on the sitewise detection of positive selection. Mol Biol Evol 29:1125-1139.

Loytynoja A, Vilella AJ, Goldman N. 2012. Accurate extension of multiple sequence alignments using a phylogeny-aware graph algorithm. Bioinformatics 28:1684-1691.

Luo C, Tsementzi D, Kyrpides N, Read T, Konstantinidis KT. 2012. Direct Comparisons of Illumina vs. Roche 454 Sequencing Technologies on the Same Microbial Community DNA Sample. PLoS ONE 7:e30087.

Mallick S, Gnerre S, Muller P, Reich D. 2009. The difficulty of avoiding false positives in genome scans for natural selection. Genome Res 19:922-933.

Markova-Raina P, Petrov D. 2011. High sensitivity to aligner and high rate of false positives in the estimates of positive selection in the 12 *Drosophila* genomes. Genome Res 21:863-874.

Ometto L, Shoemaker D, Ross KG, Keller L. 2011. Evolution of Gene Expression in Fire Ants: The Effects of Developmental Stage, Caste, and Species. Mol Biol Evol 28:1381-1392.

Penn O, Privman E, Landan G, Graur D, Pupko T. 2010. An alignment confidence score capturing robustness to guide tree uncertainty. Mol Biol Evol 27:1759-1767.

Privman E, Penn O, Pupko T. 2012. Improving the performance of positive selection inference by filtering unreliable alignment regions. Mol Biol Evol 29:1-5.

Ratnakumar A, Mousset S, Glémin S, Berglund J, Galtier N, Duret L, Webster MT. 2010. Detecting positive selection within genomes: the problem of biased gene conversion. Philos Trans R Soc Lond B Biol Sci 365:2571-2580.

Schneider A, Souvorov A, Sabath N, Landan G, Gonnet GH, Graur D. 2009. Estimates of Positive Darwinian Selection Are Inflated by Errors in Sequencing, Annotation, and Alignment. Genome Biol Evol 1:114-118.

Simola DF, Wissler L, Donahue G, et al. 2013. Social insect genomes exhibit dramatic evolution in gene composition and regulation while preserving regulatory features linked to sociality. Genome Res 23:1235-1247.

Studer RA, Penel S, Duret L, Robinson-Rechavi M. 2008. Pervasive positive selection on duplicated and nonduplicated vertebrate protein coding genes. Genome Res 18:1393-1402.

Studer RA, Robinson-Rechavi M. 2009. How confident can we be that orthologs are similar, but paralogs differ? Trends Genet 25:210-216.

Woodard SH, Fischman BJ, Venkat A, Hudson ME, Varala K, Cameron SA, Clark AG, Robinson GE. 2011. Genes involved in convergent evolution of eusociality in bees. Proc Natl Acad Sci USA 108:7472-7477.

Yang Z, dos Reis M. 2011. Statistical Properties of the Branch-Site Test of Positive Selection. Mol Biol Evol 28:1217-1228.

Yang Z, Nielsen R, Goldman N, Pedersen A-MK. 2000. Codon-Substitution Models for Heterogeneous Selection Pressure at Amino Acid Sites. Genetics 155:431-449.
